# dMyc-dependent upregulation of CD98 amino acid transporters is required for *Drosophila* brain tumor growth

**DOI:** 10.1007/s00018-022-04668-6

**Published:** 2023-01-06

**Authors:** Ana R. Rebelo, Catarina C. F. Homem

**Affiliations:** iNOVA4Health, NOVA Medical School, Faculdade de Ciências Médicas, NMS, FCM, Universidade Nova de Lisboa, Lisbon, Portugal

**Keywords:** Cancer, Neural stem cell, Amino acid transporters, CD98 heavy chain, Myc, l-amino acid transporters (LATs)

## Abstract

Tumor cells have an increased demand for nutrients to sustain their growth, but how these increased metabolic needs are ensured or how this influences tumor formation and progression remains unclear. To unravel tumor metabolic dependencies, particularly from extracellular metabolites, we have analyzed the role of plasma membrane metabolic transporters in *Drosophila* brain tumors. Using a well-established neural stem cell-derived tumor model, caused by *brat* knockdown, we have found that 13 plasma membrane metabolic transporters, including amino acid, carbohydrate and monocarboxylate transporters, are upregulated in tumors and are required for tumor growth. We identified CD98hc and several of the light chains with which it can form heterodimeric amino acid transporters, as crucial players in *brat* RNAi (*brat* ^IR^) tumor progression. Knockdown of these components of CD98 heterodimers caused a dramatic reduction in tumor growth. Our data also reveal that the oncogene dMyc is required and sufficient for the upregulation of CD98 transporter subunits in these tumors. Furthermore, tumor-upregulated *dmyc* and CD98 transporters orchestrate the overactivation of the growth-promoting signaling pathway TOR, forming a core growth regulatory network to support *brat *^IR^ tumor progression. Our findings highlight the important link between oncogenes, metabolism, and signaling pathways in the regulation of tumor growth and allow for a better understanding of the mechanisms necessary for tumor progression.

**Supplementary Information:**

The online version contains supplementary material available at 10.1007/s00018-022-04668-6.

## Introduction

The high proliferation rate characteristic of tumor cells is normally accompanied by the rewiring of metabolism, energy production pathways, and growth-promoting signals [1]. Changes in metabolism are thought to sustain the increased nutritional requirements of tumors, not only to fuel bioenergetic pathways, but also to synthesize macromolecules (DNA, RNA, proteins and lipids) [2]. Interestingly, highly proliferative tumor cells have been shown to increase the use of existing nutrients from their environment, a less energy consuming process in relation to de novo synthesis [3]. However, how the transport of extracellular nutrients into tumor cells is regulated and its implications for tumor progression are still unclear.

While many internal and external factors can influence metabolism, tumor metabolic reprogramming can be directly regulated by oncogenes, tumor suppressors or transcription factors. These transcriptional regulators not only change the expression levels of many metabolic enzymes, but also of metabolic transporters [4]. The modulation of metabolic transporters at the plasma membrane is particularly interesting, as these transporters allow the influx/efflux of many molecules from extracellular sources into the cell [5]. Indeed, some metabolic transporters have been reported to be upregulated in tumors. For instance, glucose transporters (GLUTs) are upregulated in several cancers to increase glucose uptake and sustain high rates of glycolysis (the Warburg effect) [6, 7]. However, we still lack knowledge on the contribution of different types of metabolic transporters in tumor progression. Since mammalian tumors have a high physiological complexity and several genetic limitations, it becomes technically difficult to perform in vivo studies to assess the contribution of individual metabolic transporters for tumor progression. To tackle this problem, we took advantage of *Drosophila*, a genetically powerful model with lower levels of gene redundancy, where you can induce simpler neural stem cells (NSCs)-derived tumors. *Drosophila* larval NSCs, the neuroblasts (NBs), divide to self-renew and form neurons and glia in stereotypical lineages [8]. However, mutations in tumor suppressor genes, such as *brain tumor* (*brat*) lead to the malignant transformation of NBs to tumor NBs (tNBs) and constitute a well-documented tumor model [9]. *brat* mutant tNBs are immortal and proliferate indefinitely, giving rise to massive brain tumors, which are transplantable lethal and metabolic heterogeneous [8, 10, 11]. In a tumor context tNBs suffer a metabolic reprogramming that promotes malignancy and triggers irreversible immortalization of these tNBs [11]. This indicates that metabolic changes can strongly influence tumorigenesis in tNBs. It remains, however, unknown whether tNBs require changes in the influx/efflux of certain metabolites to sustain these metabolic changes and promote tumor growth. Understanding how plasma membrane metabolic transporters regulate this process and contribute to brain tumor progression can help unravel novel tumor vulnerabilities and nutritional dependencies.

Here, we demonstrate that plasma membrane metabolic transporters are important mediators of tNB proliferation and brain tumor growth in *Drosophila*. Our study shows that the knockdown of the tumor suppressor gene *brat* with interfering RNA (IR) (*brat* ^IR^) in NBs, a well-documented tumor model, leads to the upregulation of 13 plasma membrane metabolic transporters, most of which are essential for brain tumor growth. From these transporters we identify the heterodimeric amino acid subunits CD98hc and several of its light chains as potent mediators of tumor progression. We also reveal that the oncogene dMyc, which is dysregulated in *brat* ^IR^ tumors, is involved in the upregulation of CD98hc and its light chains, Juvenile hormone Inducible-21 (JhI-21) and minidiscs (mnd), which combined lead to TOR activation to ultimately promote tumor growth. Our findings unravel that the modulation of a wide spectrum of metabolic transporters occurs in *Drosophila* brain tumors and contributes to tumor growth. Furthermore, this study highlights how the upregulation of amino acid transporters is dependent on the oncogene dMyc to sustain tumor growth and progression, contributing to the understanding of how oncogenes and metabolic transporters are connected to promote tumorigenesis.

## Materials and methods

### Fly strains and crosses

The following fly strains were used: RNAi lines used for the screen were obtained from the Vienna *Drosophila* RNAi center (VDRC, www.vdrc.at) and the Transgenic RNAi Project (TriP) lines from the Bloomington Stock Center. RNAi lines used for the screen where the following: *Scsalpha1* ^IR^ (107164KK); *CG30394* ^IR^ (3470GD); *CG5535* ^IR^ (107030KK); *CG1607* ^IR^ (BL57747); *JhI-21* ^IR^ (BL41706), *mnd* ^IR^ (42585GD), *path* ^IR^ (BL64029), *CD98hc* ^IR^ (BL57746), *Eaat1* ^IR^ (BL43287); *CG10960* ^IR^ (BL34598); *tret1-1* ^IR^ (BL42880); *MFS3* ^IR^ (107656KK), *chk* ^IR^ (37139GD), *hrm* ^IR^ (BL52902). Other RNAi lines: *dmyc* ^IR^ (106066KK); *Tor* ^IR^ (BL35578). Control RNAi lines were *mCherry* ^IR^ (BL35785) and *Luciferase (Luc)* ^IR^ (BL35788). PntGal4, UAS-myr::GFP (recombinant generated in the lab) or worGal4, AseGal80; UAS-CD8::GFP were used as type II NB drivers; UAS-Dicer2/hs-hid;inscGal4, UAS-CD8::GFP was used as a type I and type II NB driver [12]. For the transporter screen, the following transgenic line was generated: UAS-*brat* ^IR^/CyOGal80; PntGal4, UAS-myr::GFP.

### Fly rearing and dissection

For the RNAi-mediated metabolic transporter screen, UAS-*brat* ^IR^/CyOGal80; PntGal4, UAS-myr::GFP flies were crossed to UAS-candidate RNAi at 29 °C. These fly crosses were set in apple juice agar-plates for 10–15 h at 29 °C. Then, L1 (first larval instar) larvae were collected every 3 h and transferred to yeast-enriched food at 29 °C. A maximum of 100 larvae were added to each plate to avoid overcrowding. At approximately 72 h ALH (after larval hatching), at L3 stage (third larval instar), animals were collected for brain dissection.

For CD98hc depletion along tumor development, we took advantage of the temperature sensitive mutant Gal80^ts^. Briefly, UAS-*brat* ^IR^/CyOGal80; PntGal4, UAS-myr::GFP flies were crossed to *CD98hc* ^IR^; TubGal80^ts^ or TubGal80^ts^; *Luc* ^IR^ at 18 ºC. To enable temporary controlled expression of *CD98hc* ^IR^, larvae were reared at 18 °C (inactive Gal4) and shifted to 29 °C (active Gal4) for 24 h, 48 h, 72 h or 96 h until reaching wandering L3 stage when they were collected for brain dissection. At 18 ºC, Gal80^ts^ is active and prevents Gal4 transactivation, however, at 29 °C, it becomes inactive and allows Gal4-dependent transgene expression [13].

Adult fly brains were dissected approximately 24 h after adult eclosion.

### Immunohistochemistry

All brains were dissected in 1 × phosphate-buffered saline (PBS) (Invitrogen), fixed for 30 min in 4% paraformaldehyde (PFA) (Sigma), washed with PBT (PBS 1x, 0,1% Triton X-100 (Sigma)) at room temperature (RT) and incubated for 20 min–1 h with blocking solution (1 × PBS, 0,5% Triton X-100, 1% normal goat serum—NGS (Jackson ImmunoResearch)) at RT. Brains were then incubated with primary antibody diluted in blocking solution overnight at 4 °C (2 days at 4 °C for adult brains). The samples were then washed three times and incubated with blocking solution for 20 min–1 h at RT. Next, the brains were incubated with secondary antibody diluted in blocking solution for 2 h at RT (1 day at 4 °C for adult brains), protected from light. After washing three times with PBT and a final wash in PBS for 20 min, brains were mounted in a slide with aquapolymount (Polysciences, Inc) and stored at 4 °C.

### Antibodies used

The following antibodies were used: Rabbit anti-Miranda (1:1000, gift from Juergen Knoblich), mouse anti-Phospho-Histone H3 (PH3) (1:1000, Merck Millipore); rabbit anti-Phospho-4E-BP1 (Thr37/46) #2855 (1:100, Cell Signaling), mouse anti-dMyc P4C4-B1 (1:200, Developmental Studies Hybridoma Bank—DSHB). Alexa-conjugated secondary antibodies used: Alexa fluor 405, Alexa fluor 568, Alexa fluor 647 (1:1000, Molecular probes).

### Brain dissociation and cell sorting

L3 larvae were dissected in Schneider’s medium (Sigma). After dissection, brains were transferred to supplemented Schneider’s medium (10% fetal bovine serum (Sigma), 20 mM Glutamine (Sigma), 0.04 mg/mL l-Glutathione (Sigma), 0.02 mg/mL Insulin (Sigma) Schneider’s medium (Sigma)) and washed once. Then, brains were enzymatically dissociated with supplemented Schneider’s medium with 1 mg/mL Papain (Sigma) and 1 mg/mL Collagenase I (Sigma) for 1 h at 30 °C. Afterwards, brains were washed twice with supplemented Schneider’s medium and mechanically disrupted in supplemented Schneider’s medium using a pipette tip. The cell suspension was filtered through a 30 μL mesh into a 5 mL FACS tube (BD Falcon) and immediately sorted by fluorescence activated cell sorting (FACS) (FACS Aria III, BD). GFP positive NBs and tNBs were collected directly into TRIzol LS (Invitrogen™) to preserve RNA and stored at − 80 °C until further processing.

### RNA extraction and qPCR

mRNA from sorted NBs and tNBs was isolated using TRIzol™ LS Reagent (Invitrogen™) according to the manufacturer’s instructions, adjusted for a small amount of RNA. Then RNA was treated with TURBO DNA-free™ Kit (Invitrogen™) and cDNA was prepared using the RevertAid First Strand cDNA Synthesis Kit (Thermo Scientific™).

The following primers were used for amplification:

- *dpn*—fw: CGCTATGTAAGCCAAATGGATGG; rv: CTATTGGCACACTGGTTAAGATGG
- *Act5C*—fw: GATAATGATGATGGTGTGCAGG; rv: AGTGGTGGAAGTTTGGAGTG
- *dmyc—*fw: GTGGACGATGGTCCCAATTT; rv: GGGATTTGTGGGTAGCTTCTT
- *brat—*fw: TGGAAACTCGGACCAGAATC*;* rv: ATGGAAGCGAAGAACTGGTG
- *CD98hc—*fw*:* TGGAAACCCTGGCTACTTTG; rv: ATCTTGTCCGGCAGATTGTC
- *JhI-21—*fw: ACCGATATGCCAATGGAGTG; rv: AATCTTCTCCTCGCCATCAG
- *mnd*—fw: GATCAACCTGTGGTGCTCCA; rv: GGCCCTCTCTTCCCAAAGTC
- *CG30394*—fw: GCCAGTCTCTATTCGCTGCT; rv: AATCGAAAGCGTTGTTCAGG
- *CG5535*—fw: TGTGACCATTGGCGAGTTTA; rv: GGTGCTTAAACCCTTGACGA
- *CG1607*—fw: GTCATTTCCGGCCTTTTCTC; rv: TAATCAGCACCCGACTTGGT
- *path*—fw: CGGTCTCATCATGGGAATCT; rv: CTTGTGTCCGCATTTTACCA
- *Eaat1*—fw: CCCAGACACGCTTATGGATT; rv: TAGATCTCAGTGCGGTGCTG
- *CG10960*—fw: CTGCGGAATCAATGCTGTTA; rv: CTACCAGGGTGGAGACGAAG
- *Tret1-1*[14]-fw:ATGTCTCCGACATCGCCATGGTTC; rv: TCACCCATCATCAGCCAGGGAATG
- *Chk*—fw: TATCTGGGGGATCTGTCCTG; rv: GACCATCAGGCCCATGTAGT
- *hrm*—fw: CTCTCCTTCTGGGCATCATC; rv: TTCGCAAAATACGACGTCAC

qPCRs were performed using GoTaq qPCR Master mix (Promega) on a QuantStudio™ 5 Real-Time PCR System (Applied Biosystems™). The expression of all genes was normalized to *Act5C* and relative mRNA levels were calculated against the control (*brat* ^IR^; *mCherry* ^IR^ for tNBs and *mCherry* ^IR^ for NBs) using the 2^(−ΔΔCt)^ method [15]. All experiments were done with technical triplicates.

To quantify the efficiency of the RNAi lines used, each RNAi line was crossed to ActinGal4, an ubiquitous driver, and mRNA was extracted from whole brains, cDNA was prepared using the RevertAid First Strand cDNA Synthesis Kit (Thermo Scientific™) and levels were analyzed by qPCR. RNAi of CG10906 (BL34598) in whole animals under the control of ActinGal4 was lethal, for this reason RNAi of CG10906 was instead crossed with InscGal4, a NB specific driver, mRNA was extracted and cDNA prepared from these brains. RNAi of CD98hc (BL57746) under the control of either InscGal4 or AseGal4, NB specific drivers, was lethal and thus this line was not analyzed by RT-qPCR.

### Image acquisition and phenotype analysis

Immunofluorescent Z-stack images from whole brain or single lobe images from the posterior side were acquired using a confocal microscope LSM880 (Carl Zeiss). For volume measurements, stacks were acquired throughout the whole brain with 1 μm interval between each slice. Images were processed using the image analysis software FIJI and volume was measure by generating a 3D reconstruction of the GFP^+^ positive volume of brains in Imaris. The quantification of PH3^+^ NB number in tumors was done using the microscopy image analysis software Imaris (Oxford instruments) and normalized for the volume of tumor quantified. To measure P-4E-BP1 and dMyc fluorescence intensity confocal images were acquired with identical laser power, scan settings and adjustments. Mean fluorescence intensity was measured using FIJI by manually selecting the GFP^+^ area in a representative focal plane and measuring the mean gray value in each region of interest. Then, the corrected fluorescence intensity was obtained by removing the average mean gray value of the background.

### Quantification of branched chain amino acids (BCAA)

UAS-*mcherry* ^IR^ (control), UAS*-JhI-21* ^IR^ and UAS-*mnd* ^IR^ were crossed with ActinGal4 and their adult progeny was analyzed. For each genotype 10 two-day adult flies were flash-frozen in liquid nitrogen and homogenized in 200 mL of assay buffer. Homogenates were centrifuged for 10 min at 12.000 rpm and 10 mL of supernatant used to measure BCAA levels (kit K564-100Biovision, now Abcam ab83374). Color intensity was quantified using a microplate reader (Biotek Synergy HT Microplate Reader). Technical duplicates were included for each sample in each plate. A “blank” and a “background” well were also included in every plate for sample normalization as recommended by the manufacture’s protocol. Protein quantification was performed for sample amount normalization using BCA protein quantification assay (BCA Assay, ThermoFisher) according to the manufacture’s protocol. Three biological experiments were performed.

### Statistical analysis

When analyzing multiple genotypes, p-values were obtained with One-Way ANOVA for multiple comparisons. Otherwise, unpaired two‐tailed Student's *t*‐test was used to assess statistical significance between two genotypes. All data are expressed as mean ± SEM.

## Results

### ***brat***^IR^ tNBs upregulate plasma membrane metabolic transporters which are important for tumor growth

Currently, it is well established that metabolic rewiring can be a critical step for tumor development and progression (reviewed in Ref. [16]). We hypothesized that the metabolic changes occurring in *Drosophila* tumor cells [11] could translate into different demands for extracellular nutrients. To test this hypothesis, we took advantage of the available transcriptomes of isolated *brat* ^IR^ tNBs and wild-type type II NBs, the subtype of NBs from where these tumors are formed [17], and examined the differential expression of plasma membrane metabolic transporters. We selected candidate metabolic transporter genes in the FlyBase annotation gene set, according to their Gene Ontology (GO) terms. Genes were selected using GO molecular function “transmembrane transporter activity” (GO:0022857). We identified 774 genes from *Drosophila melanogaster* annotated with this term, which were then subjected to a narrower selection by molecular function, in particular: amino acid transmembrane transporter activity—GO:0015171 and GO:0003333 (64 annotated genes); carbohydrate transmembrane transporter activity—GO:0015144 (36 annotated genes); monocarboxylic acid transmembrane transporter activity—GO:0008028 (31 annotated genes); amide transmembrane transporter activity—GO:1904680 (8 annotated genes); amine transmembrane transporter activity—GO:0005275 (3 annotated genes); nucleobase-containing compound transmembrane transporter activity—GO:0015932 (23 annotated genes) and azole transmembrane transporter activity—GO:1901474 (5 annotated genes) (Table S1). Since transmembrane transporters can be localized in different cellular membranes, such as mitochondria and lysosome membranes, from these genes, we excluded the ones that were shown/predicted not to be localized in the plasma membrane (33 excluded genes) (Table S1—genes highlighted in grey). We have then selected significantly upregulated transporters in tNBs in relation to wild-type NBs according to the transcriptome data (Table 1; Table S1—candidates highlighted in green). Based on the values of Fragments Per Kilobase of transcript per Million (FPKM) mapped reads, a metric of relative expression in RNA sequencing, we selected genes that were upregulated, i.e. that increased their FPKM in tNBs in relation to NBs (*p* value < 0.05). We considered a threshold of FPKM > 4 in tNBs as an arbitrary cut-off to exclude genes that are little expressed in both genotypes.

**Table 1:** Upregulated metabolic transporters in *brat* ^IR^ tNBs, selected according to the available transcriptome data (Landskron et al. 2018 [17]) and GO molecular functions

Gene	log_2_FoldChange	Adjusted p-value	FPKM-NB	FPKM-tNBbrat	GO Molecular Function
Amino acid transporters (61 annotations GO:0015171)					
*SLC38 Family of sodium-coupled neutral amino acid transporters (Total members: 2 genes)*					
CG30394	1.12	0.03	0.95	6.80	Sodium-coupled neutral amino acid transporters
*SLC7 Family of amino acid transporters (Total members: 11 genes)*					
CG5535	3.39	2.18e-29	5.49	69.69	Cationic amino acid transporter
CG1607	2.88	3.93e-06	1.8	57.63	L-type amino acid transporter
JhI-21	1.78	2.19e-09	27.96	178.43	L-type amino acid transporter
mnd	2.88	1.76e-05	4.81	94.73	L-type amino acid transporter
*SLC36 Family of proton-coupled amino acid transporters (Total members: 10 genes)*					
Path	2.95	9.95e-06	11.03	282.52	Proton-coupled amino acid transmembrane transporter
*SLC3 Family of heterodimeric amino acid ancillary subunits (Total members: 1 gene)*					
CD98hc	0.66	0.03	139.26	336.7	Heavy chain of heteromeric amino acid transporters
*SLC1 Family of glutamate and neutral amino acid transporters (Total members: 2 genes)*					
Eaat1	1.10	1.0e-3	2.76	21.41	High-affinity glutamate transporter
Carbohydrate transporters (22 annotations GO:0015144)					
*SLC2 Family of Hexose sugar transporters Total members: 25 genes)*					
CG10960	4.45	4.01e-42	0.34	20.21	Sugar transporter activity
Tret1-1	1.37	1.98e-05	8.51	75.62	Trehalose transporter activity
*SLC17 Family of organic anion transporters (Total members: 25 genes)*					
MFS3	5.35	2.37e-06	1.14	71.97	Glucose and trehalose transporter activity
Monocarboxylate transporters (31 annotations GO:0008028)					
SLC16 Family of monocarboxylate transporters (Total members: 14 genes)					
Chk	4.04	4.36e-27	0.43	24.03	Monocarboxylic acid transporter activity
Hrm	0.90	5.4e-3	1.33	8.65	

All gene symbols according to Flybase: flybase.org

*FPKM* fragments per kilobase of transcript per million, *SLC*solute carrier

From this analysis we concluded that *brat* ^IR^ tNBs selectively upregulated some amino acid, carbohydrate and monocarboxylate transporters, but did not display upregulation of plasma membrane metabolic transporters from the remaining GO categories listed above. tNBs increased the expression of 8 amino acid transporters, 3 sugar transporters and 2 monocarboxylate transporters (Table 1), all of which belong to the solute carrier (SLC) superfamily, the largest group of transporters widely known to be implicated in tumorigenesis [18]. Furthermore, this analysis shows that *Drosophila* tumors, like mammalian tumors, also have the capacity to reprogram the expression of several metabolic transporters.

**Fig. 1:**
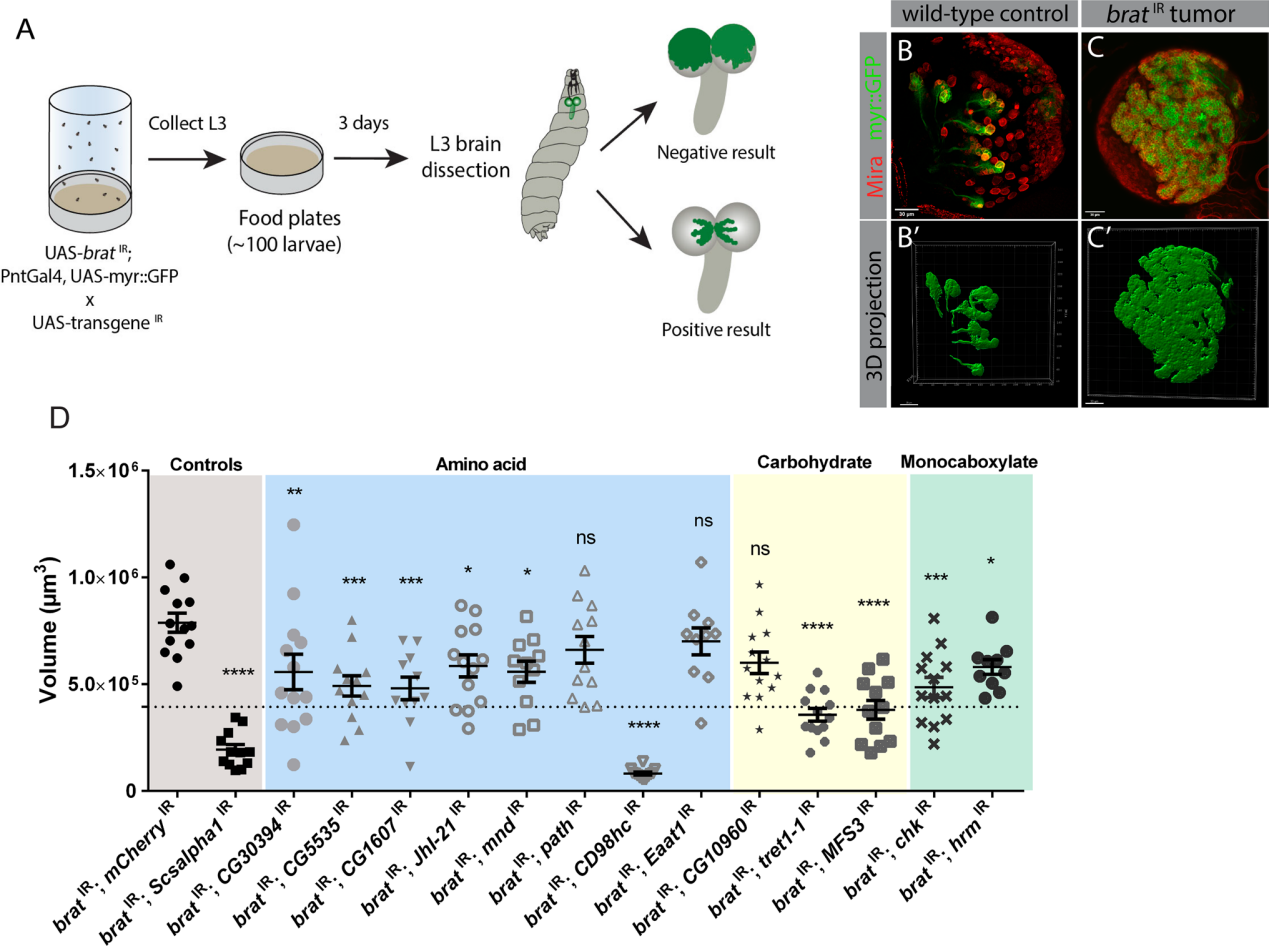
Upregulated metabolic transporters contribute for brain tumor growth. **A** Experimental design of the plasma membrane metabolic transporter screen in tumors. **B**, **C** Fixed L3 brains expressing myristoylated::GFP (membrane bound GFP, myr::GFP) and the indicated transgenes under the control of type II NB driver PntGal4. Brains labelled with an antibody against Miranda (Mira), a NB marker. **B** *mCherry* ^IR^, UAS-myr::GFP (wild-type control); **C** *brat* ^IR^, UAS-myr::GFP (*brat* ^IR^ tumor). Mira, red; myr::GFP, green. **B’**, **C’** Corresponding Imaris 3D projections of GFP expressing cells. Scale bars represent 30 μm. **D** Quantification of the GFP^+^ volume of L3 larval brain tumors with depletion of the respective metabolic transporter (UAS-*brat* ^IR^; PntGal4, UAS-myr::GFP x UAS-transporter ^IR^). Negative control was *brat* ^IR^; *mCherry* ^IR^ and positive control *brat* ^IR^; *Scsalpha1* ^IR^. Dashed line represents 50% of the average of *brat* ^IR^ *mCherry* ^IR^ tumor volume. Error bars represent ± SEM; *n* ≥ 10 brains. Significance for each candidate RNAi compared with control tumors (*brat* ^IR^, *mCherry*.^IR^) using a one-way ANOVA with post-hoc Dunnett’s multiple comparisons test. *P value < 0.05; **P value < 0.01; ***P value < 0.001; ****P value < 0.0001. *ns* non-significant (*P* value ≥ 0.05)

We next hypothesized that these transporters might have an important role in the growth of *Drosophila* brain tumors. To investigate the biological relevance of this transcriptional change in tumor progression we devised a genetic screen, where we aimed at depleting upregulated metabolic transporters in tNBs and analyzed how this impacts tumor growth. Taking advantage of UAS-RNAi transgenes [19] we individually knocked down the upregulated transporters listed in Table 1 specifically in *brat* ^IR^ tumors (UAS-*brat* ^IR^; PntGal4, UAS-myr::GFP x UAS- RNAi candidate transporter) and measured tumor volume. To ensure that the animals had the same nutrient availability and less variability in tumor size, we designed an experimental protocol to rear the same number of larvae for each genotype and to synchronize larval development (Fig. 1A). We dissected larval brains approximately 72 h ALH, at L3 stage, and analyzed tumor volume using Imaris (Bitplane). A representation of the 3D projections of *brat* ^IR^ tumors (membrane myr-GFP labels type II NB lineages, Miranda (Mira) is a NB marker) and its’ comparison to wild-type type II NB lineages obtained with Imaris is shown in Fig. 1B, C. The knockdown of the metabolic enzyme succinyl coenzyme A synthetase-alpha subunit 1 (Scsalpha1) was used as a positive control, as it has been previously described to reduce tumors and rescue *brat* ^IR^ tumor-induced lethality [11]. As a negative control we used the knockdown of *mCherry*, a gene that is not normally expressed in *Drosophila*. As expected, knockdown of *Scsalpha1* led to a striking reduction in tumor volume, a good indicator that the screen was working (Fig. 1D).

Overall, our quantitative analysis showed that, compared to control tumors (*brat* ^IR^*; mCherry* ^IR^), depleting upregulated metabolic transporters led to a significant reduction in tumor volume, except for the amino acid transporter path, Eaat1, and the carbohydrate transporter CG10960 (Fig. 1D). This suggests that the transport of amino acids, carbohydrate and monocarboxylates in *brat* ^IR^ tNBs plays an important role in tumor growth. Interestingly, out of 13 transporters tested, depletion of CD98hc, the heavy chain required for several essential amino acid transporter complexes, the trehalose transporters Tret1-1 and the glucose and trehalose transporter MFS3 reduced average tumor volume above 50% (Fig. 1D, candidates whose mean volume is below the dashed line). We have additionally evaluated the knock down efficiency of the RNAi lines used by RT-qPCR (Fig. S1A). *CD98hc* ^IR^ caused animal lethality even when expressed only in type I central brain NBs, confirming the efficiency of the RNAi but precluding quantitative evaluation of *CD98hc* knock down in whole brains by RT-qPCR. *Chk* ^IR^ has been previously characterized [20].

Taken together, our data shows that most of the transcriptionally upregulated metabolic transporters are essential for tumor growth. This suggests that the import/export of certain amino acids, sugars and monocarboxylates is important for tumor progression.

### CD98 heterodimeric amino acid transporter family is crucial for *brat*^IR^ tumor growth

**Fig. 2:**
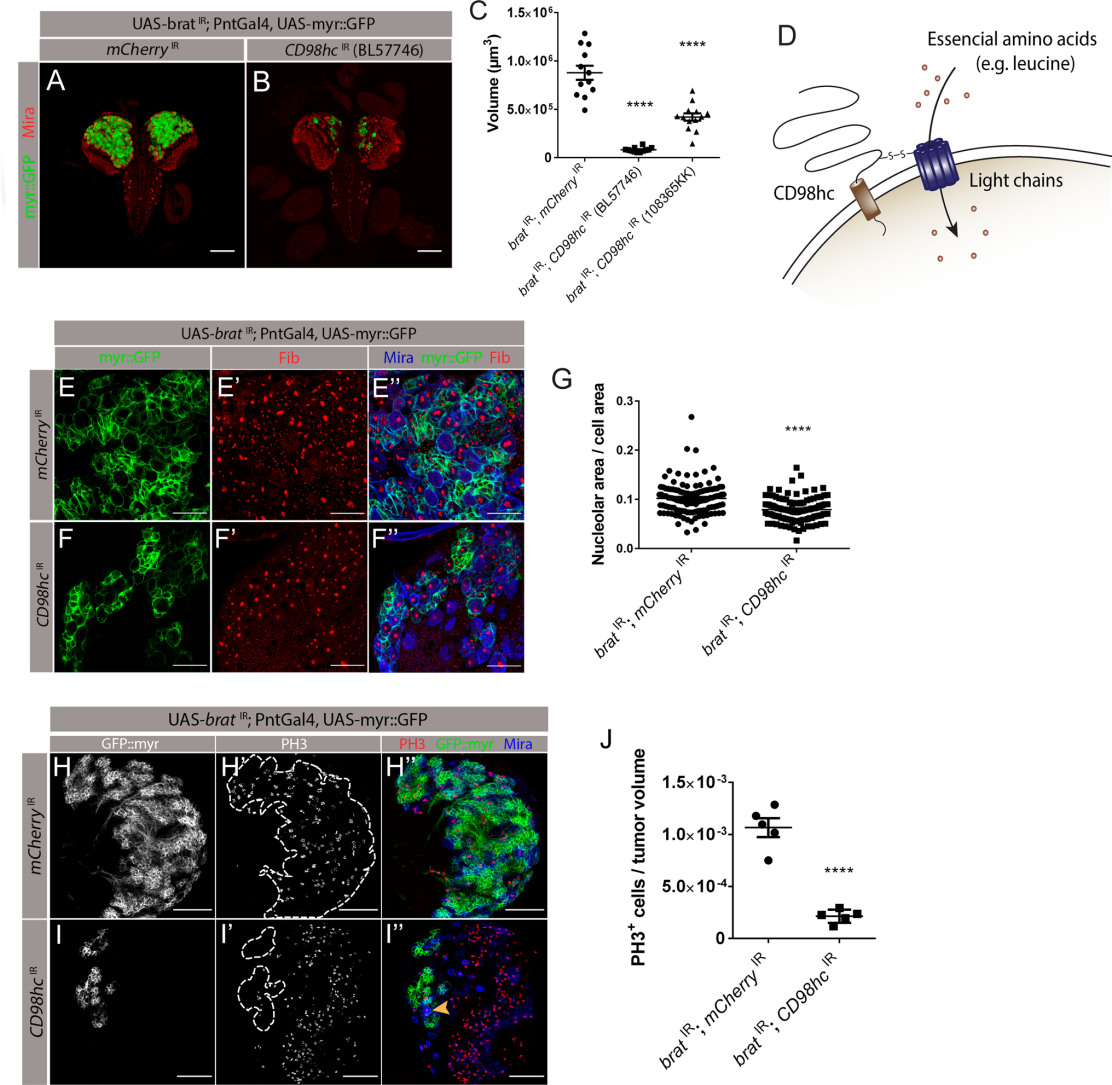
Knockdown of *CD98hc* prevents tumor overgrowth. **A**, **B** Fixed L3 brains expressing myr::GFP and the indicated transgenes under the control of type II NB driver PntGal4. **A** *brat* ^IR^; *mCherry* ^IR^. **B** *brat* ^IR^*; CD98hc* ^IR^. Scale bars represent 100 μm. red, Miranda (Mira); green, myr::GFP. **C** Quantification of the tumor volume with two independent RNAi lines against *CD98hc*. Significance for each RNAi compared with control tumors (*brat* ^IR^; *mCherry* ^IR^) using a one-way ANOVA with post-hoc Dunnett’s multiple comparisons test. ****P value < 0.0001. **D** Cartoon depicting CD98 heterodimeric amino acid transporter structure. **E**, **F** L3 brains of indicated genotypes with the nucleolus labeled by fibrillarin antibody in red; green, myr::GFP; blue, Mira. Scale bars represent 20 μm. **E**–**E’’** *brat* ^IR^*; mCherry* ^IR^ tNBs. **F**–**F’’** *brat* ^IR^; *CD98hc* ^IR^ tNBs. **G** Quantification of the ratio of nucleolar area per cell area in *brat* ^IR^; *mCherry* ^IR^ and *brat* ^IR^; *CD98hc* ^IR^ tNBs. **H,** **I** L3 tumors of the indicated genotypes labelled with PH3. **H**–**H’’’** *brat* ^IR^; *mCherry* ^IR^ tumors. **I**–**I’’’** *brat* ^IR^*; CD98hc* ^IR^ tumors. red, PH3; green, myr::GFP; blue, Mira. Dashed outlines represent GFP positive tumor area and yellow arrow points to a PH3 positive NB. Scale bars represent 50 μm. **J** Quantification of PH3 positive cells per tumor volume of indicated genotypes. Statistical analysis was done using unpaired two-tailed *t* test; ****P value < 0.0001. All error bars represent ± SEM

Recently, several studies have shown that amino acids have a pivotal role in cancer progression, being involved in many functions from biosynthetic support to signaling regulation [21]. Consistently, we noticed that knockdown of the amino acid transporter heavy chain CD98 (CD98hc) practically abrogated the growth of *brat* ^IR^ tumors, reducing average tumor volume by 90% (Figs. 1D and  2A–C). To validate the CD98hc RNAi-induced phenotype obtained, we tested a second CD98hc RNAi line, which also led to significantly smaller tumors, although not as efficiently as the first RNAi line (Fig. 2C). Neither RNAi lines have predicted off targets (flybase.com), thus we decided to pursue this work using the RNAi line that presented the stronger phenotype (BL57746).

CD98 heterodimeric complex amino acid transporters, are disulphide-bound heterodimers that promote the intake of leucine and other bulky hydrophobic amino acids. Each CD98 heterodimer is composed by a transmembrane heavy chain (CD98hc) that covalently binds to a multipass transmembrane light chain that confers substrate specificity [22] (Fig. 2D). Since CD98hc can be a part of several transporter complexes that uptake essential amino acids, we hypothesized that *brat* ^IR^ *CD98hc* ^IR^ tumors were smaller due to a reduction in the amino acid intake in tNBs. Amino acid deprivation can be characterized by a variety of cellular features, including a reduction in the area of the nucleolus, the major cellular site for ribosome production [23, 24]. Hence, to analyze whether CD98hc-depleted tumors might be suffering from a reduction in amino acid availability, we evaluated the nucleolar area of tNBs. For this, we labelled nucleoli with the marker Fibrillarin and measured the nucleolar size as the ratio between nucleolar area and cellular area as previously described [25] (Fig. 2E–G). We found the nucleolar size in *brat* ^IR^ *mCherry* ^IR^ control tNBs to be significantly larger in relation to *brat* ^IR^ *CD98hc* ^IR^ tNBs (Fig. 2G), suggesting that *CD98hc* knock-down might indeed translate into reduced amino acid intake in tNBs.

As a reduction in nucleolar size can also correlate with a reduction in cellular proliferation and growth [24], we next tested whether these tumors were smaller due to a reduction in their proliferative capacity. For this, we analyzed the pattern of phosphohistone H3 (PH3) positive cells, a marker for cells in mitosis, by counting the total number of PH3^+^ cells normalized to the respective tumor volume (Fig. 2H–J). This analysis showed that CD98hc-depleted *brat* ^IR^ tNBs were still dividing, shown by the presence of PH3^+^ cells (Fig. 2I’’, yellow arrow), although with a significantly reduced mitotic index (Fig. 2J). This significant reduction in the mitotic rate of *CD98hc* ^IR^ tumors could thus account for one of the mechanisms by which CD98hc depletion might reduce tumor volume.

**Fig. 3:**
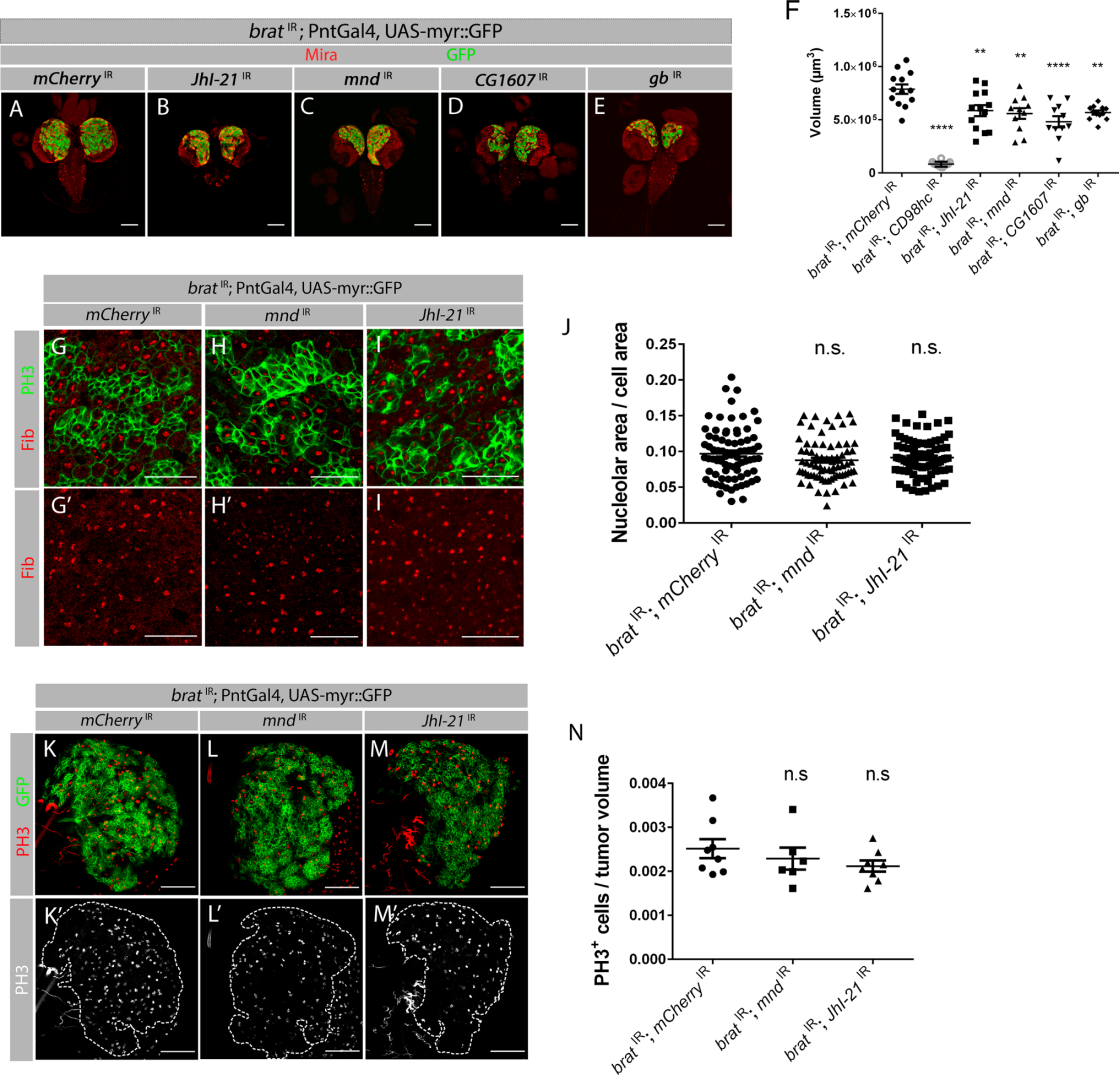
Knock down of CD98 light chains significantly reduce *brat* ^IR^ tumor volume. **A**–**E** *brat* ^IR^ tumors with individual knockdown of the indicated light chain under the control of type II NB driver PntGal4. **A** Control brain tumors *brat* ^IR^; *mCherry* ^IR^*.* **B** *brat* ^IR^; *JhI-21* ^IR^ (BL41706). **C** *brat* ^IR^*; mnd* ^IR^ (42585GD). **D** *brat* ^IR^*; CG1607* ^IR^ (BL57747). **E** *brat* ^IR^*; gb* ^IR^ (1262GD). Scale bars represent 100 μm. **F** Quantification of the tumor volumes with the respective light chain depletion in *brat *^*IR*^ tumors. Error bars represent ± SEM. Significance for each candidate RNAi compared with control tumors (*brat* ^IR^ *mCherry* ^IR^) using a one-way ANOVA with post-hoc Dunnett’s multiple comparisons test. **P value < 0.01; ****P value < 0.0001. **G**–**I** L3 brains of indicated genotypes with the nucleolus labeled by fibrillarin antibody in red; green, myr::GFP. Scale bars represent 20 μm. **J** Quantification of the ratio of nucleolar area per cell area. **K**–**M** L3 tumors of the indicated genotypes labelled with PH3. red, PH3; green, myr::GFP. Dashed outlines represent GFP positive tumor area. Scale bars represent 50 μm. **N** Quantification of PH3 positive cells per tumor volume of indicated genotypes. Statistical analysis was done using one-way ANOVA with post-hoc Dunnett’s multiple comparisons test.; *ns* non-significant (*P* value ≥ 0.05). All error bars represent ± SEM

*Drosophila* has been shown to have a single functional CD98hc orthologue and five predicted amino acid transporters that share a significant degree of homology with the human light chains LAT1 and LAT2: Juvenile hormone Inducible-21 (JhI-21), minidiscs (mnd), CG1607, genderblind (gb) and Sobremesa (Sbm) [26]. Expression analysis of *sbm* in the transcriptome data revealed that it has negligible expression in both NBs and tNBs and, therefore, this gene was not further studied (FPKM-NB *sbm* = 0.02; FPKM-*brat* ^IR^ tNB *sbm* = 0.13; *p* value > 0.05). From the remaining 4 light chains, only *CG1607*, *JhI-21* and *mnd* were significantly upregulated in tNBs based on the transcriptome (Table 1—SLC7 family of transporters), and their knockdown led to a significant reduction in *brat* ^IR^ tumors (Figs. 1D and 3A–D, F; Fig. S1C). As it is not clear if light chains need to be upregulated to exert an important function in tumors, we decided to also test the role of gb, which was not upregulated in tumors (FPKM-NB *gb* = 4.89; FPKM-*brat* ^IR^ tNB *gb* = 12.14; *p* value > 0.05). Consistently *gb* knockdown in *brat* ^IR^ tumors also led to a significant reduction in tumor volume (Fig. 3E, F). These results suggest that all the CD98 light chains expressed in NBs have an important role in *brat* ^IR^ tumor growth. To better understand the mechanism by which light chains affect tumor growth, we focused on the light chains JhI-21 and mnd as examples, as these two proteins were experimentally shown to be involved in leucine transport [26–28]. We started by confirming their upregulation in tumors by RT-qPCR (Fig. S1B). Next we asked if their knock down affects amino acid availability or NB proliferation capacity as observed for *CD98hc* ^IR^. For this we labelled nucleoli with the marker Fibrillarin as described above and measured the nucleolar size as the ratio between nucleolar area and cellular area (Fig. 3G–J). We found that the nucleolar size did not change significantly in tumors with *JhI-21* and *mnd* knock-down (Fig. 3J). We next tested whether these tumors were smaller due to a reduction in their proliferative capacity by analyzing the number of PH3^+^ cells normalized to the respective tumor volume. This analysis showed that the knock down of light chains *JhI-21* or *mnd* in *brat* ^IR^ tNBs does not prevent NB division and the differences in mitotic index were not significant (Fig. 3K–N). As the tumor rescue induced by *brat* ^IR^ *JhI-21* ^IR^ and *brat* ^IR^ *mnd* ^IR^ is not as big as the one caused by *CD98hc*^*IR*^ (Fig. S1C; Fig. 3F), PH3 and Fibrillarin might not be sensitive enough to measure subtle changes in mitotic index and nucleolar area, respectively. Alternatively, the light chains might have more stage specific roles during tumor growth and thus the analysis at 3^rd^ instar larval stages might have missed their larger effect. Consistently it has been previously shown that JhI-21 is differentially expressed in the brain in early L3 vs. late L3 stages [29].

As there are several light chains, and it has been previously reported that different light chains can compensate for each other [26], we next asked if this might also be happening in the brain. To test this, we have analyzed how the knock down of light chains mnd and JhI-21 affect each other. This analysis surprisingly revealed that the sole knock down of *mnd* leads to a significant reduction in the transcript levels of *JhI-21* (Fig. S1D). Interestingly another study has also reported that knockdown of *mnd* can lead to a decrease in JhI-21 protein levels in the brain [30]. On the other hand, knock down of *JhI-21* only causes a 20% reduction in the levels of *JhI-21* and does not cause significant changes in the levels of *mnd* (Fig. S1D). Although the knock down levels of *JhI-21* are not very high, this line (BL41706) has been shown to be efficient in other studies [30, 31].

### CD98hc depletion does not prevent tumor initiation but inhibits *brat*^IR^ tumor growth

**Fig. 4:**
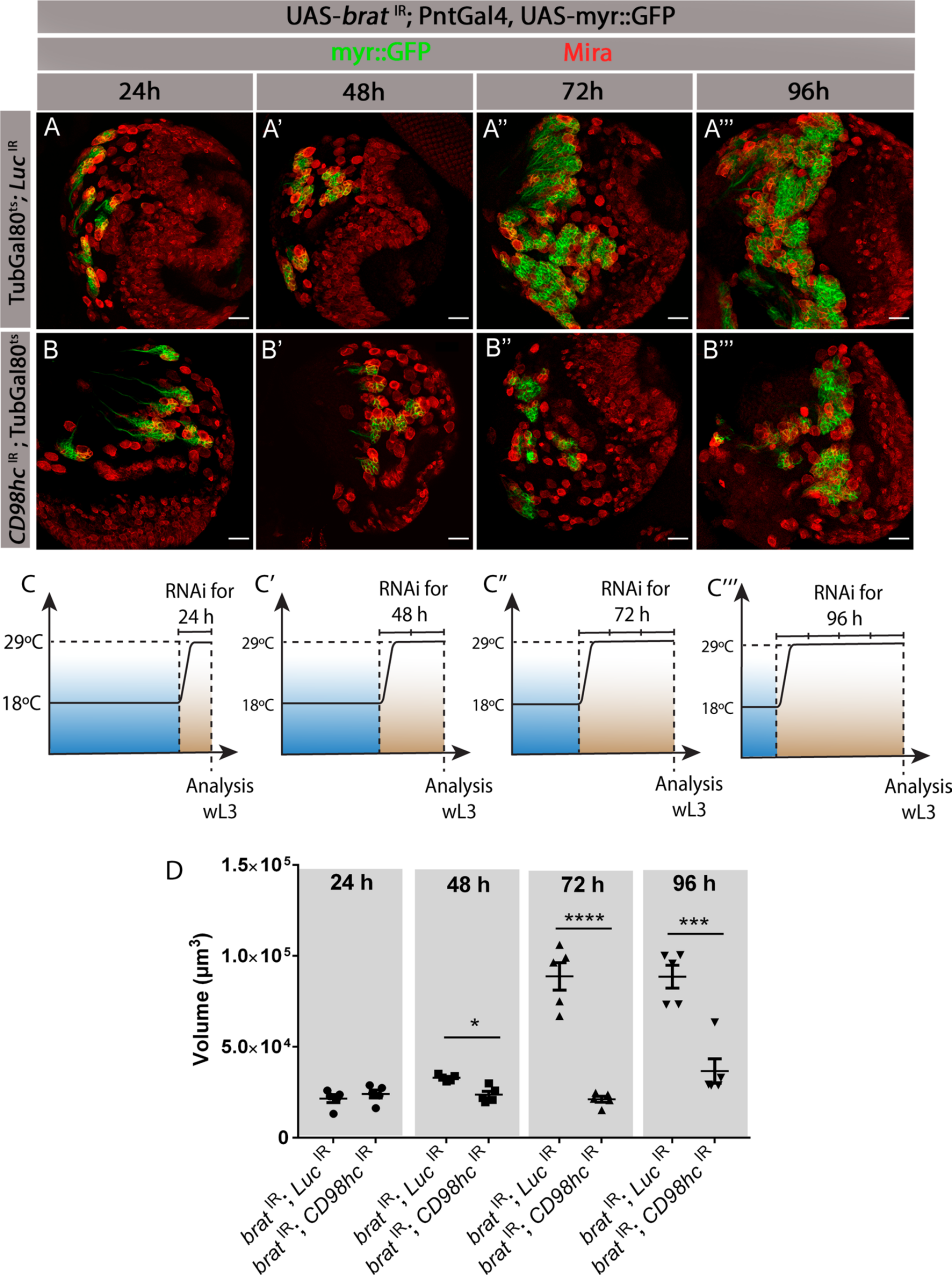
CD98hc depletion does not avoid tumor formation but leads to *brat* ^IR^ tumor reduction. **A**, **B** Fixed L3 brain lobes expressing myr::GFP and the indicated transgenes under the control of type II NB driver PntGal4 at the indicated time points after tumor induction. **A**–**A’’’** *brat* ^IR^*; Luc* ^IR^ control tumors, **B**–**B’’’** *brat* ^IR^*; CD98hc*.^IR^ tumors. Red, Miranda (Mira); green myr::GFP, Scale bars represent 20 μm. **C** Scheme depicting the experimental setup. Larvae were reared at 18 °C, preventing the expression of the RNAis, and shifted to 29 °C to induce RNAi expression for: **C** 24 h; **C’** 48 h; **C’’** 72 h and **C’’’** 96 h. **D** Quantification of the tumor volume for the indicated genotypes with RNAi expression during the indicated time periods. Statistical analysis was done using unpaired two-tailed *t* test. *P value < 0.05; ***P value < 0.001; ****P value < 0.0001

Since CD98 is crucial for the correct intake of essential amino acids, which are an important part of cellular homeostasis [32], we asked whether CD98hc depletion could also lead to the disappearance/death of some of the original 8 type II NBs (per lobe), this way reducing the number of cells capable of originating the tumor and, thus, indirectly preventing tumor formation. To test this possibility, we knocked down *CD98hc* and *brat* in a time-controlled manner and analyzed how the tumor is initiated in this condition. For this, we took advantage of the temperature sensitive Gal4/Gal80^ts^ system [13]. Gal80^ts^ enables the temporal control of the Gal4/UAS system, thus, under permissive conditions (18 ºC) Gal80 blocks the expression of *brat* and *CD98hc* RNAis. Then, by shifting the animals to 29 ºC, Gal80 becomes inactive and enables RNAi expression for any timeframe desired. Hence, we ubiquitously expressed Gal80^ts^ (TubGal80^ts^) in larvae, reared the animals at 18 ºC and then shifted larvae to 29 ºC to induce *brat* and *CD98hc* RNAis expression in NBs for 24 h, 48 h, 72 h and 96 h (Fig. 4A–C). *brat* ^IR^; *Luc* ^IR^ were used as control tumors (*Luc*-Luciferase is a non-*Drosophila* gene). As previously described [11], in a control situation (TubGal80^ts^; *brat* ^IR^*; Luc* ^IR^), there was no tumor formation in the first 24 h of RNAi induction (Fig. 4A, compare to wild-type lineages in Fig. 1B), and only after ~ 48 h of *brat* depletion type II NB lineages become disorganized and start to overproliferate (Fig. 4A’) with tumors quickly growing after this point (Fig. 4A’–A’’’). Consistently, upon CD98hc knockdown (*brat* ^IR^*; CD98hc* ^IR^; TubGal80^ts^) tumors were still absent at 24 h of RNAi induction and the number and morphology of type II NB lineages were similar to a control situation (Fig. 4B compare to 4 A and Fig. 1B). After 48 h of Brat and CD98hc depletion, we started to observe increased number of NBs and lineage disorganization similar to what is observed during tumor initiation, although the tumor volume is smaller than in *brat* ^IR^ control tumors (Fig. 4B’, compare to 4A’, Fig. 4D). At later timepoints (72 h and 96 h post RNAi induction), CD98hc-depleted tumors do not grow to the same extent as control tumors and the tumor volume is even more significantly reduced (Fig. 4B’’, B’’’, compare to A’’ and A’’’, Fig. 4D). Therefore, *brat* ^IR^ and *CD98hc* ^IR^ tumors initiate from the same number of type II NBs and do so with similar timings although their growth afterwards is significantly smaller compared to control tumors.

Another feature that is involved in tumor growth and characterizes tNBs is their immortality and capacity to proliferate into adulthood [33], in contrast to wild-type NBs that usually exit cell cycle and are decommissioned during pupal development (± 16 h after puparium formation—APF for type II NBs) [34, 35]. As knock down of CD98hc decreases the growth potential of Brat-deleted tumors, we then hypothesized that CD98hc depletion in *brat* ^IR^ tNBs could also avert the immortal proliferative potential of tNBs. Thus, we analyzed whether CD98hc-depleted tNBs were still present at later developmental stages, namely in newly ecloded adults, a stage where wild-type NBs have normally already decommissioned. As expected, in adult wild-type brains, there were no NBs present (Fig. S2 A *n* = 5). However, *brat* ^IR^; *CD98hc* ^IR^ tNBs were still present in some adult brains (Fig. S2 B, GFP labelled tNBs present in 3 brains out of 5) and were mitotically active (Fig. S2 B’, PH3^+^ cells inside the tumor outline—yellow dashed line). This shows that, CD98hc knockdown does not prevent tNBs immortal proliferative potential, as they can persist into adulthood.

To further confirm if the effect we observe upon CD98 subunits’ depletion is specific to tumors, we also knocked down *CD98hc* and the light chains *JhI-21, mnd, CG1607* and *gb* in non-transformed wild-type type II NB lineages. Although NBs were still present, depletion of CD98hc resulted in alterations in the stereotypical morphology of their lineages (Fig. S3 B, compare to A) and a reduction in NB number (Fig. S3 G). On the other hand, knockdown of the light chains in wild-type lineages led to no morphological defects in NB lineages nor alteration in the number of NBs (Fig. S3 C–F; G). To test if knockdown of CD98hc and its light chains might be causing a reduction in amino acid availability or proliferation rate of NBs in a non-tumor context, we evaluated the nucleolar area and mitotic rate of NBs knocked down for each of these genes in an otherwise wild-type background. The nucleolar size was not affected by *JhI-21* ^IR^ and *mnd* ^IR^ (Fig. S3 H), while *CD98hc* ^IR^ caused a significant reduction in nucleolar area when compared to wild-type NBs (Fig. S3 H). As the CD98 transporter complex is known to mediate branched chained amino acid (BCAA) uptake (such as Valine, Leucine and Isoleucine) [22], to further confirm if knock down of CD98 transporters affect amino acid transport we quantified total levels of BCAAs using a biochemical colorimetric kit (K564-100Biovision, now Abcam ab83374) in animals knocked down for these transporter subunits. We expressed UAS-RNAi under the control of the ubiquitous promoter actinGal4 and analyzed adult flies. Knock down of *CD98hc* in whole animals was lethal and therefore we could not analyze these animals further. We then observed that *mnd* knock-down leads to a significant decrease in BCAA levels, although *JhI-21* knockdown did not cause a significant difference in the amount of BCAA in whole animals (Fig. S3 I). As the RNAi line used to knockdown *JhI-21* is not very efficient (Fig. S1 A, D) and the line used to knockdown *mnd* causes a large reduction in the levels of both *mnd* and *JhI-21* (Fig. S1 D) this could explain the difference in the results obtained for both lines. Overall, these results allow us to suggest that the knock down of *mnd* and *JhI-21* affect amino acid transport.

Furthermore, the mitotic rate was not affected when the light chains *JhI-21* or *mnd* were knocked down in wild-type NBs, in contrasting *CD98hc* ^IR^, which caused a significant reduction in NB proliferation rate (Fig. S3 J). Although knockdown of *CD98hc* led to a reduction in the proliferative rate of NBs in a wild-type background, it does not impair normal NB fate or lineage formation in contrast to the dramatic reduction in tNB number observed when *CD98hc* is knocked down in a tumor background. Together these data suggest that CD98hc heterodimer transporters are particularly important in the tumor context.

### dMyc is required for the upregulation of CD98hc and light chains JhI-21 and mnd

**Fig. 5:**
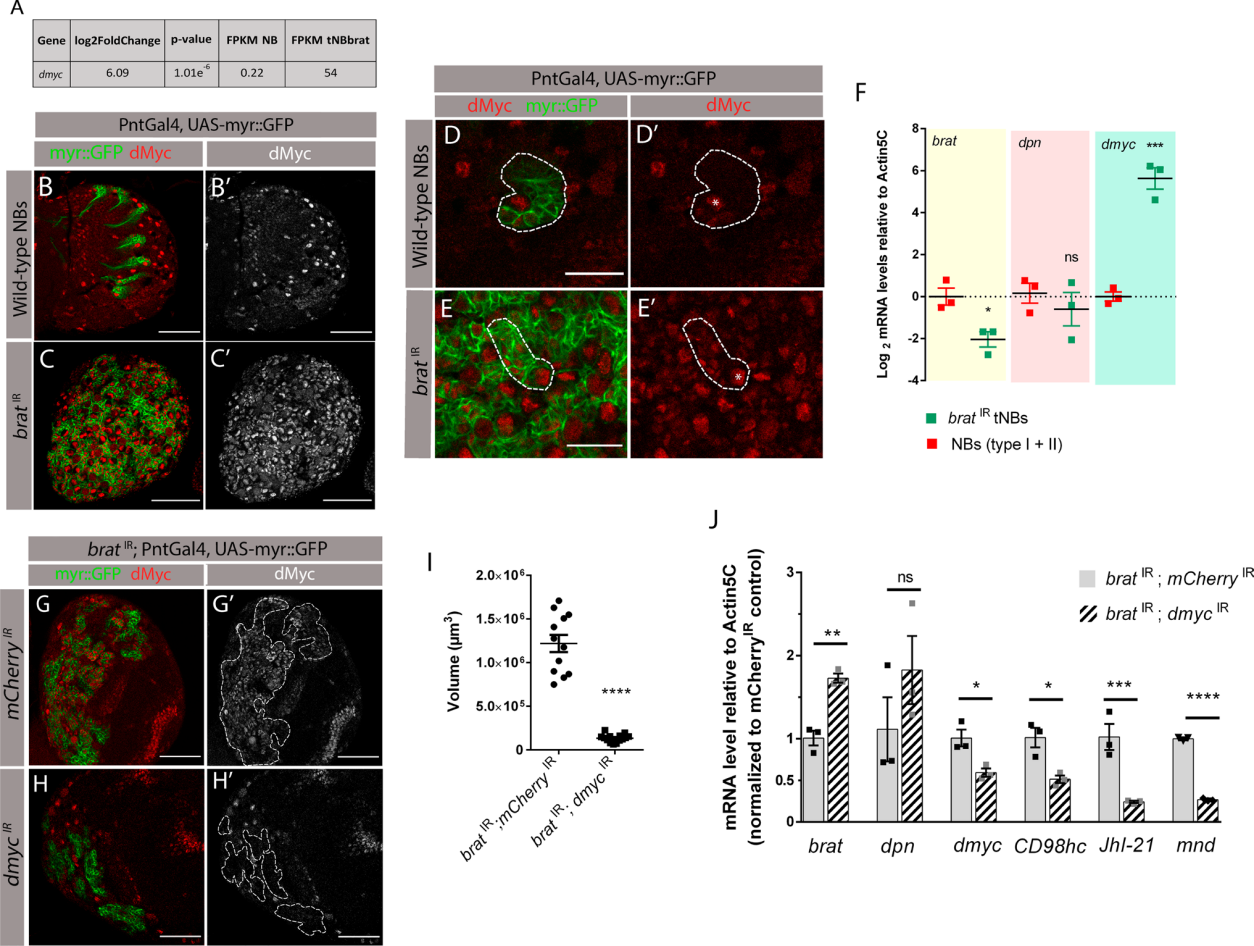
dMyc is required for the upregulation of CD98 heterodimeric amino acid transporters in *brat* ^IR^ tumors. **A** Expression value of *dmyc* in FPKM according to the transcriptome data of Landskron et al. (2018) [17]. FPKM—Fragments Per Kilobase of transcript per Million. **B**–**E** Fixed L3 brain lobes expressing myr::GFP under the control of type II NB driver PntGal4 with dMyc staining. **B**–**B’** control wild-type type II NB lineages (PntGal4, UAS-myr::GFP*/mCherry* ^IR^). **C**–**C’** *brat* ^IR^ tumors (*brat* ^IR^; PntGal4, UAS-myr::GFP). **D**–**D’** Close-up of a wild-type type II lineage (NB marked with an asterisk and respective lineage inside the dashed line) and **E**–**E’** *brat* ^IR^ tumor (“primary” tNB marked with an asterisk and respective progenitors inside the dashed line). **F** qPCR of *brat*, *deadpan (dpn)* and *dmyc* in wild-type NBs (type I + type II) vs*. brat* ^IR^; *mCherry* ^IR^ tNBs. Deadpan, a NB marker, was included as a control. **G**, **H** L3 brain lobes of indicated genotypes. **G**, **G’** control tumors *brat* ^IR^*; mCherry* ^IR^. **H**, **H’** *brat* ^IR^*; dmyc* ^IR^ tumors. Dashed lines represent the tumor outline. red/white, dMyc; green, myr::GFP. Scale bars represent 50 μm (**B**, **C** and **G**, **H**) and 20 μm (**D**, **E**). **I** Quantification of the tumor volumes for the indicated genotypes. **J** qPCR of *brat*, *dpn*, *dmyc*, *CD98hc*, *JhI-21* and *mnd* in control tumors (*brat* ^IR^; *mCherry* ^IR^) vs*.* dMyc-depleted tumors (*brat* ^IR^; *dmyc* ^IR^). All RT-qPCR data shown represents the mean (± SEM) of triplicates. Statistical analysis was done using unpaired two-tailed *t* test. *P value < 0.05; **P value < 0.01; ***P value < 0.001; ****P value < 0.0001. *ns* non-significant (*P* value ≥ 0.05)

Our previous results show that CD98-dependent amino acid transport is essential for *brat* ^IR^ tumor growth. The upregulation of CD98hc and its light chains in tNBs is most probably responsible for an increase in amino acid intake that yields the proper pool of amino acids for tumor growth. However, what drives these expression changes remains unknown. Therefore, we next sought to identify the mechanism behind the transcriptional upregulation of CD98 transporter subunits in tNBs. Previous studies demonstrated that the expression of the transcription factor *Drosophila myc* (*dmyc*) is dysregulated in *brat* ^IR^ tumors [9, 36]. Its mammalian homologue, MYC, is overexpressed and/or activated in 50–60% of all cancers, promoting tumor initiation and progression [37]. In particular, MYC has been shown to play an important role in regulating metabolic reprogramming in normal development and cancer [38]. Hence, we postulated that dMyc might be involved in the upregulation of CD98 complex transporters in *brat* ^IR^ tumors to mediate the sufficient uptake of essential amino acids and support tumor growth. To test this hypothesis, we started by confirming whether dMyc is upregulated in our *brat* ^IR^ tumor model by assessing *dmyc* expression levels in both wild-type NBs and *brat* ^IR^ tNBs. The analysis of the transcriptome data available from Landskron et al. (2018) [17] showed that *dmyc* expression was increased more than 68-fold in *brat* ^IR^ tNBs (Fig. 5A). Furthermore, when analyzing dMyc protein levels with an antibody we observed that dMyc protein levels are dysregulated in *brat* ^IR^ tumors, as previously shown [9] (Fig. 5B, C). As expected, dMyc was present in wild-type NBs, but not in the differentiating daughter cells (Fig. 5D’). On the other hand, in *brat* knockdown tumor brains, dMyc was present in all tNBs (Fig. 5E’), consistent with the fact that Brat inhibits dMyc post-transcriptionally [9]. Furthermore, we confirmed by RT-qPCR that tNBs suffered an upregulation of *dmyc* in relation to wild-type NBs (Fig. 5F) [36].

To investigate whether dMyc is required for the upregulation of CD98hc and its light chains, we knocked down *dmyc* in tNBs (UAS-*brat* ^IR^; PntGal4, UAS-myr::GFP x UAS-*dmyc* ^IR^). In *dmyc* depleted tumors, dMyc protein levels were lower in relation to control tumors, showing that *dmyc* knockdown was efficient (Fig. 5G and H). Moreover, as expected, *brat* ^IR^; *dmyc* ^IR^ tumors were significantly smaller in relation to *brat* ^IR^ control tumors (Fig. 5I), which is consistent with a previous report showing that *dmyc* knockdown inhibits ectopic NB formation in *brat* mutants [39]. Then, we isolated *brat* ^IR^ tNBs and *dmyc*-depleted *brat* ^IR^ tNBs by FACS and measured gene expression levels of *dmyc*, *CD98hc* and the light chains *JhI-21* and *mnd*. To control for the different tumor sizes between each genotype, we analyzed the same number of tNBs for each condition and normalized the expression values against a standard qPCR reference gene. This way, we ensured that any differential gene expression obtained is not a consequence of a reduction in tNB number. First, we confirmed by RT-qPCR that *dmyc* RNAi effectively reduced *dmyc* expression in tNBs (Fig. 5J). We have also included in our RT-qPCR control primers to detect *deadpan* (*dpn*), a NB marker, and *brat* to confirm knock down by the RNAi transgene. Interestingly, *dmyc*-depleted *brat* ^IR^ tNBs displayed a significant downregulation of both *CD98hc* and the light chains *JhI-21* and *mnd* in relation to control tumors (Fig. 5J). This data shows that dMyc is indeed required for the upregulation of the CD98 heterodimeric complex transporters in *brat* ^IR^ tumors, confirming our initial hypothesis.

Surprisingly, we noticed *brat* levels were significantly increased in *brat* ^IR^,*dmyc* ^IR^ tNBs. To test if the observed rescue in tumor growth by *dmyc* knockdown could be a consequence of the increase in expression of *brat,* we have quantified the levels of Brat protein, using a specific antibody in *brat* ^IR^ tumors vs. *brat* ^IR^, *dmyc* ^IR^ tumors. We measured the mean fluorescence intensity of Brat in wild-type NB lineages, in *brat* ^IR^ and in *brat* ^IR^*, dmyc* ^IR^ tumors. As expected, we observed a significant reduction in Brat levels in *brat* ^IR^*, mcherry* ^IR^ tumors in relation to wild-type (Fig. S4 A, B, D). There was, however, no significant difference in Brat levels between *brat* ^IR^*, mcherry* ^IR^ and *brat* ^IR^*,dmyc* ^IR^ tumors (Fig. S4 B, C, D). This analysis thus showed that there is no significant increase in the levels of Brat protein when *dmyc* is depleted in *brat* ^IR^ tumors and the increase in *brat* mRNA levels detected by RT-qPCR likely has no functional significance.

Altogether, our results confirm that dMyc is involved, either directly or indirectly, in the transcriptional upregulation of heterodimeric amino acid transporters in tumors.

### dMyc is involved in the activation of TOR signaling in *brat*^IR^ tumors

Essential amino acids, such as leucine and isoleucine are not only essential for biosynthetic purposes, but also for activating the target of rapamycin (TOR) pathway [40]. TOR signaling can sense amino acid availability and induce protein synthesis and this pathway is usually overactivated in several cancers, as it can regulate cell proliferation and survival [41]. Since we show that dMyc influences the expression of the essential amino acid transporter members *CD98hc*, *JhI-21* and *mnd*, we postulated that this could ultimately lead to TOR activation in *brat* ^IR^ tumors which would then contribute to the cascade of events that result in tumor growth.

**Fig. 6:**
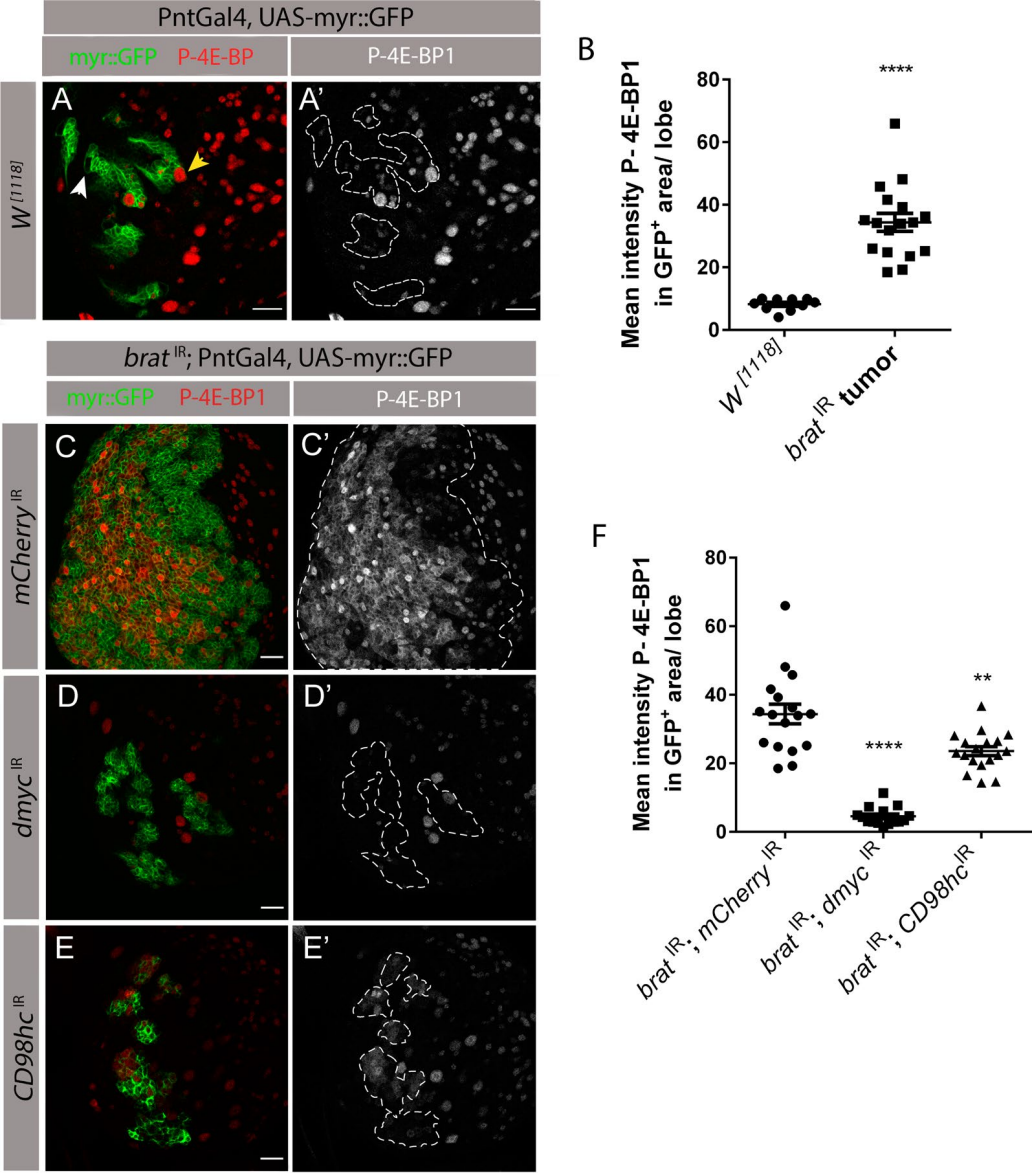
dMyc is involved in TOR activation in *brat* ^IR^ tumors. **A**, **C**–**E** Fixed L3 brain lobes expressing myr::GFP and the indicated transgenes under the control of type II NB driver PntGal4. **A**–**A’** Phosphorylated 4E-BP1 (P-4E-BP1) staining in wild-type type II NB lineages. Dashed line outlines type II NB lineages, yellow arrow points to a P-4E-BP1 positive NBs and white arrow points to a P-4E-BP1 negative NB. **B** Quantification of the mean fluorescence intensity of P-4E-BP1 in the GFP^+^ wild-type lineages vs GFP^+^ tumor area (*brat* ^IR^ tumors). **C**–**E** L3 brain lobes of indicated genotypes with P-4E-BP1 staining. **C**–**C’** *brat* ^IR^*; mCherry* ^IR^ control tumors. **D**–**D’** *brat* ^IR^; *dmyc* ^IR^. **E**–**E’** *brat* ^IR^; *CD98hc* ^IR^. Dashed line represents the respective GFP ^+^ tumor outline. **F** Quantification of the mean intensity of P-4E-BP1 in the GFP^+^ *brat* ^IR^ tumor area in relation to *brat* ^IR^; *CD98hc* ^IR^ and *brat* ^IR^; *dmyc* ^IR^ tumors. Error bars represent ± SEM. All statistical analysis were done using unpaired two-tailed *t* test; **P value < 0.01; ****P value < 0.0001. Red, P-4E-BP1; green, GFP. All scale bars represent 20 μm

To test this hypothesis, we first asked if TOR levels are increased in a tumor context. We have thus measured TOR activation levels by examining the levels of phosphorylated 4E-BP1 (P-4E-BP1) in tumors and wild-type NB lineages. In its unphosphorylated form, 4E-BP1 inhibits translation by binding to the eukaryotic initiation factor 4E (eIF4E). When TOR is activated, TOR complex 1 (TORC1) directly phosphorylates 4E-BP1 and blocks its ability to regulate eIF4E, thus, allowing for translation initiation [42]. As the volume of wild-type type II NB lineages and *brat* ^IR^ tumors are very different, to allow for an unbiased comparison we measured the mean fluorescence intensity of P-4E-BP1 in these two genotypes. In wild-type NB lineages we detected P-4E-BP1 in a sub-set of type II NBs (Fig. 6A, P-4E-BP1^+^ NB—yellow arrow; P-4E-BP1^−^ NB—white arrow) and in a small number of progeny cells in the lineage. However, in *brat* ^IR^ tumors, there was a significant increase in the mean levels of P-4E-BP1 in relation to wild-type NB lineages (Fig. 6A–C). Hence, TOR activity is increased in a tumor context.

Next, to understand whether dMyc and the consequent upregulation of CD98 transporters are required for the overactivation of TOR in tumors, we tested how knock down of *dmyc* or *CD98hc* affects TOR activation in tumors. We have, therefore, again measured P-4E-BP1 mean fluorescence intensity to control for the different tumor sizes obtained in each genotype. Consistent with our hypothesis, *brat* ^IR^*; dmyc* ^IR^ tumors, as well as *brat* ^IR^; *CD98hc* ^IR^ tumors were characterized by a significant decrease in P-4E-BP1 mean fluorescence intensity in relation to *brat* ^IR^*; mCherry* ^IR^ control tumors (Fig. 6C–F), reflecting a decrease in TOR activity. This suggests that dMyc and CD98hc are required to over-activate TOR signaling in a tumor context.

In some tissues dMyc has been reported to act downstream of the TOR pathway to control growth in *Drosophila* [43]. Hence, we wondered if TOR could also be involved in the upregulation of dMyc levels in tumors this way promoting a tumor activation loop. To answer this question, we measured the mean fluorescence intensity of dMyc in *brat* ^IR^ tNBs when knocked down for *CD98hc* or *Tor* (part of the TORC1 complex) (Fig. S5 A–C). This analysis revealed that dMyc levels did not suffer any significant change between these three genotypes (Fig. S5 D), which suggests that increased TOR signaling or essential amino acid uptake do not increase dMyc levels in *brat* ^IR^ tumors. Interestingly, we noted that Tor-depleted *brat* ^IR^ tumors were significantly smaller in relation to control tumors (Fig. S5 E), which is consistent with our previous results indicating that TOR signaling has an important role in tumor progression (Fig. 6C–F).

## Discussion

To support rapid cellular metabolism, tumors often increase the influx of metabolites by upregulating plasma membrane metabolic transporters to increase nutrient uptake, but how this is regulated and how it affects tumor progression is still not clear [16]. In this study, we show that *Drosophila brat* ^IR^ NB-derived brain tumors upregulate 13 plasma membrane metabolic transporters, including amino acid, carbohydrate and monocarboxylate transporters, and that most of them are important for proper tumor growth. This suggests that *Drosophila* tumors, like mammalian tumors, can upregulate selected plasma membrane metabolic transporters to support tumor bioenergetics and biosynthesis. Although the knock-down of some metabolic transporters did not lead to tumor size reduction, we cannot exclude that the RNAis used might not work. Alternatively, some of these transporters might be important only in small sub-sets of *brat* ^IR^ tNBs, which would be consistent with previous observations showing that these tumors can have a high metabolic heterogeneity [11]. There might also be some compensatory transcriptional rewiring in tumor cells that compensate for the loss of a particular metabolite, for instance through the increase in the expression of a similar transporter [26, 44].

One of the tumor-relevant transporters identified in this study is the CD98 heterodimeric amino acid transporter family, comprised by CD98hc and several light chains. Individual knockdown of these genes revealed that they are particularly important players in *Drosophila* brain tumor progression, as their knockdown causes a dramatic reduction in tumor size. Indeed, we showed that *brat* ^IR^ *CD98hc* ^IR^ tumors suffered a reduction in both tNB nucleolar size and tumor mitotic index, known to reflect amino acid deprivation and a reduction in proliferation, respectively. Interestingly, the importance of CD98 complex amino acid transporters for tumor proliferation is in line with a previous study reporting that exogenous amino acids are the major source of carbon in proliferating mammalian cells, contrary to the rapidly consumed glucose and glutamine [45]. CD98hc seems to be particularly important for *brat* ^IR^ tumors, as its depletion in wild-type does not affect NB fate and only mildly reduces NB number, proliferation and nucleolar size. Consistent with our findings, another recent study has beautifully demonstrated that CD98 light chains *JhI-21* and *mnd* are also upregulated in Ras^V12^*/scrib*^−/−^ and bantam*/rab5*^−/−^ malignant tumors and their depletion strongly reduces tumor growth [31]. This suggests that the requirement of CD98 heterodimeric amino acid transporters might be a common vulnerability in other tumor models. Furthermore, interestingly, it has been reported that CD98hc and its light chain LAT1 are often overexpressed and associated with poor prognosis in several aggressive cancers, such as non-small-cell lung cancer [46], glioma [47] and triple-negative breast cancers, underlining the important role of CD98hc in tumor progression. Interestingly in mammals CD98hc has additionally been reported to be a co-receptor of beta integrins [48, 49], but as integrin members are not expressed or expressed at negligible levels in type II NBs or in *brat*^*IR*^ NBs [17] the function of CD98hc in NB tumors seems to be integrin independent.

We have also assessed by which mechanisms *brat* ^IR^ tNBs upregulate CD98 heterodimeric complex transporters and how this modulates tumor progression. We propose that in the absence of Brat, the transcription factor dMyc is upregulated and induces the expression of CD98hc and its light chains to form heterodimeric amino acid transporters. It remains unknown whether dMyc instructs a transcriptional cascade or directly increases CD98 heterodimer complex expression. However, data from a previous study that analyzed dMyc DNA-binding sites in *Drosophila* Kc cells, has revealed that dMyc can associate with the genes *CD98hc*, *JhI-21* and *mnd*, suggesting that dMyc might directly regulate the expression of these genes [50].

Additionally, we found that *brat* ^IR^ tumors overactivate the growth promoting signaling pathway TOR, a feature that is conserved in human cancers [41]. As TOR is usually activated in the presence of leucine, our observations suggest that in *brat* ^IR^ tumors, TOR activation could be mediated by dMyc, probably through the upregulation of the leucine transporter CD98, to promote tumor growth and progression. Consistent with this, depletion of either CD98hc, dMyc or Tor strikingly reduced tumor size as well. Nevertheless, this does not exclude that dMyc could synergistically promote tumor growth via other mechanisms, since it is a known transcription factor involved in several cellular processes that lead to proliferation, growth and cell survival [38]. In some cell types, as *Drosophila* wing imaginal disc cells, it has been shown that activation of the TOR pathway activation can lead to dMyc protein accumulation [43]. However, unexpectedly, when we knocked down *Tor* and *CD98hc* in *brat* ^IR^ tumors, there were no significant changes in dMyc levels. Although we did not pursue this question further, as tNBs are stem cell-derived cancer cells and dMyc is an important factor to maintain stemness [51], there might be other compensatory mechanism regulating dMyc levels in tNBs. Furthermore, we cannot exclude that there might be subtle changes in dMyc levels with relevant physiological consequences, which might be difficult to quantify with antibody staining.

**Fig. 7:**
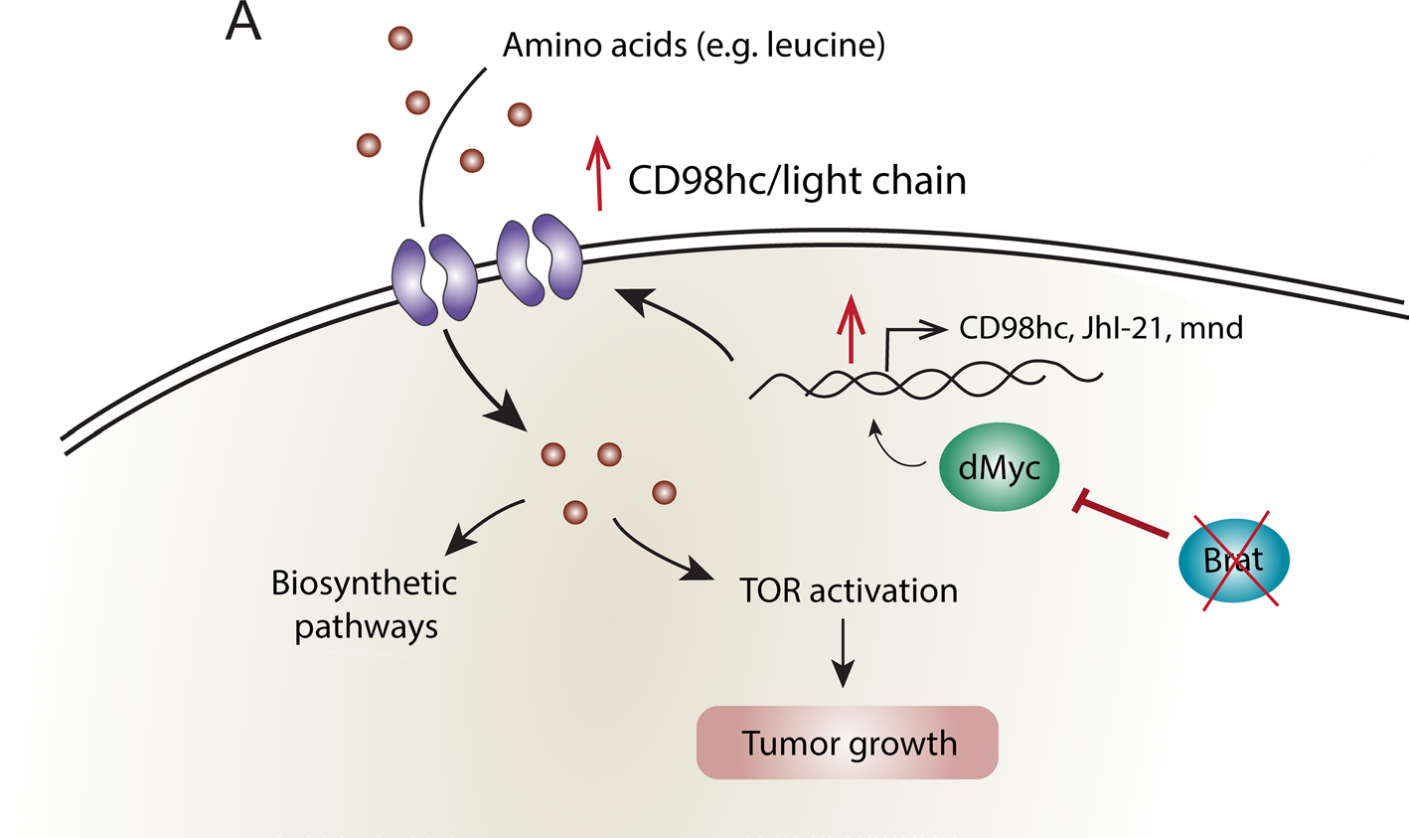
Proposed model for the integration of CD98 and dMyc upregulation with tumor growth. See text for more details

Overall, our model proposes that upon NB malignant transformation via *brat* knockdown, *dmyc* is upregulated, leads to the upregulation of *CD98hc* and light chains, which increases the influx of amino acids in tNBs. These amino acids will in turn contribute to biosynthetic pathways and increase the levels of TOR signaling activation to promote aberrant growth and survival of tNBs (Fig. 7A). In conjunction, dMyc and heterodimeric amino acid transporter upregulation as well as TOR overactivation can serve as a core growth-promoting route for *brat* ^IR^ tumors.

## Supplementary Information

Below is the link to the electronic supplementary material.

Supplementary file1 (XLSX 24 KB)

Supplementary file2 Fig. S1 CD98 light chains’ knockdown reduces tumor growth. (A) Quantification of RNAi efficiencies by qPCR. qPCR of indicated target gene in whole brains expressing indicated RNAi transgenic line under the control of actinGal4 except *CG10906* ^IR^ which was driven by inscGal4. (B) qPCR of *CD98hc*, *mnd* and *JhI-21* in wild-type type II NBs vs. *brat *^IR^ tNBs. (C) Quantification of *brat* ^IR^ tumor volumes with individual knockdown of the indicated light chain under the control of type II NB driver PntGal4 with two independent RNAi lines: *mnd *^IR^ (BL62207 and 42585GD) and *JhI-21* ^IR^ (BL41706 and 108509KK). Error bars represent ±SEM. Significance for each candidate RNAi compared with control tumors (*brat* ^IR^; *mCherry* ^IR^) using a one-way ANOVA with post-hoc Dunnett’s multiple comparisons test. ** P value < 0.01; *** P value < 0.001; **** P value < 0.0001 (D) qPCR of *mnd* and *JhI-21* in control brains (ActinGal4; *mCherry* ^IR^), *mnd* knocked down brains (ActinGal4; *mnd* ^IR^) and *JhI-21* knocked down brains (ActinGal4; *JhI-21* ^IR^). All RNAi transgenes driven under the control of ubiquitous driver ActinGal4. Whole 3rd instar larval brains were analysed. All RT-qPCR data shown represents the mean (± SEM) of triplicates. Statistical analysis was done using unpaired two-tailed t test. * P value < 0.05; ** P value < 0.01; *** P value < 0.001; **** P value < 0.0001. (TIF 58437 KB)

Supplementary file3 Fig. S2 CD98hc depletion does not prevent the immortal proliferative potential of *brat* ^IR^ tNBs. (A-B) Fixed newly ecloded adult brains expressing myr::GFP under the control of type II NB driver PntGal4. (A) Wild-type adult brain (PntGal4, UAS-myr::GFP), (B) *brat* ^IR^; *CD98hc* ^IR^ tumor (*brat* ^IR^/*CD98hc* ^IR^; PntGal4, UAS-myr::GFP). Red, PH3; green, myr::GFP. White dashed lines represent the brain outline and yellow dashed line in B’ represents the tumor outline. Scale bars represent 100 μm (TIF 2480 KB)

Supplementary file4 Fig. S3 Knockdown of *CD98hc* and light chains of CD98 heterodimeric transporters in wild-type context does not impair normal NB fate. (A-F) Fixed L3 brain lobes expressing CD8::GFP and the indicated transgenes under the control of type II NB driver WorGal4, AseGal80. (A) control type II NBs lineages. Type II NBs depleted for: (B) CD98hc, (C) JhI-21, (D) mnd, (E) CG1607 and (F) gb. Red, Miranda (Mira); green, myr::GFP. Scale bars represent 50 μm. (G) Quantification of type II NB number in 3rd instar larval brain lobes of indicated genotypes in the wild-type context. (H) Quantification of the ratio of nucleolar area per cell area of *CD98hc* ^IR^ vs. *JhI-21* ^IR^ vs. *mnd* ^IR^ all normalized against control *mCherry* ^IR^. (I) Quantification of BCAA levels of indicated RNAi lines driven by ActinGal4. *mcherry* ^IR^ = control. (J) Quantification of PH3^+^ NBs per brain volume of indicated genotypes. All statistical analysis were done using one-way ANOVA with post-hoc Dunnett’s multiple comparisons test ns – non-significant (P value ≥ 0.05); * P value < 0.05; ** P value < 0.01; *** P value < 0.001. All error bars represent ±SEM. (TIF 108558 KB)

Supplementary file5 Fig. S4 Brat protein levels are not significantly upregulated when dMyc is depleted in *brat* ^IR^ tumors. (A-C) Fixed L3 brain lobes expressing myr::GFP and the indicated transgenes under the control of type II NB driver PntGal4 stained for Brat. Red, Brat; green, myr::GFP. Outlines represent GFP positive area. Scale bars represent 50 μm. (D) Quantification of Brat mean fluorescence intensity in the GFP+ area. Statistical analysis was done using unpaired two-tailed t test; **** P value < 0.0001, ns – non-significant (P value ≥ 0.05). All error bars represent ±SEM (TIF 74578 KB)

Supplementary file6 Fig. S5 dMyc levels do not change significantly with *Tor* or *CD98hc* knock down in *brat* ^IR^ tumors. (A-C) Fixed L3 brain lobes expressing myr::GFP and the indicated transgenes under the control of type II NB driver PntGal4 stained for dMyc (red). myr::GFP, green. (A,A’) control brain tumor (*brat* ^IR^; *mCherry* ^IR^), (B,B’) *brat* ^IR^; *CD98hc* ^IR^ and (C,C’) *brat* ^IR^; *Tor* ^IR^ tumors. Dashed lines represent the respective GFP^+^ tumor outline. Scale bars represent 50 μm. (D) Quantification of dMyc mean fluorescence intensity in the GFP^+^ tumor area. Error bars represent ±SEM. Values for each genotype compared with control tumor (*mCherry* ^IR^) using a one-way ANOVA with post-hoc Dunnett’s multiple comparisons test. ns – non-significant (P value ≥ 0.05). (E) Quantification of the tumor volume of *brat* ^IR^; *Tor* ^IR^ tumors in relation to *brat* ^IR^; *mCherry* ^IR^ control tumors. Statistical analysis was done using unpaired two-tailed t test; **** P value < 0.0001 (TIF 57038 KB)

## Data Availability

The transcriptome dataset analyzed in the current study has been previously published in Landskron et al. (2018) [17] and is available at the NCBI Gene Expression Omnibus (accession no. GSE87085), https://www.ncbi.nlm.nih.gov/geo/query/acc.cgi?acc

## Abbreviations

ALH: After larval hatching
APF: After puparium formation
Brat: Brain tumor
ºC: Celsius degrees
CD98hc: CD98 heavy chain
FACS: Fluorescence activated cell sorting
FPKM: Fragments per kilobase of transcript per million
GO: Gene ontology
h: Hours
IR: Interference RNA
L1: First larval instar
L3: Third larval instar
Luc: Luciferase
min: Minutes
NB: Neuroblast
NSC: Neural stem cell
P-4E-BP: Phosphorylated 4E-binding protein
PH3: Phospho-histone 3
RNAi: RNA interference
RT: Room temperature
SLC: Solute carrier
tNB: Tumor neuroblast
