# Supplementary figures and images for: dMyc-dependent upregulation of CD98 amino acid transporters is required for Drosophila brain tumor growth
Source: Cell Mol Life Sci. 2023 Jan 6;80(1):30. doi: 10.1007/s00018-022-04668-6 (PMC9823048; doi:10.1007/s00018-022-04668-6)

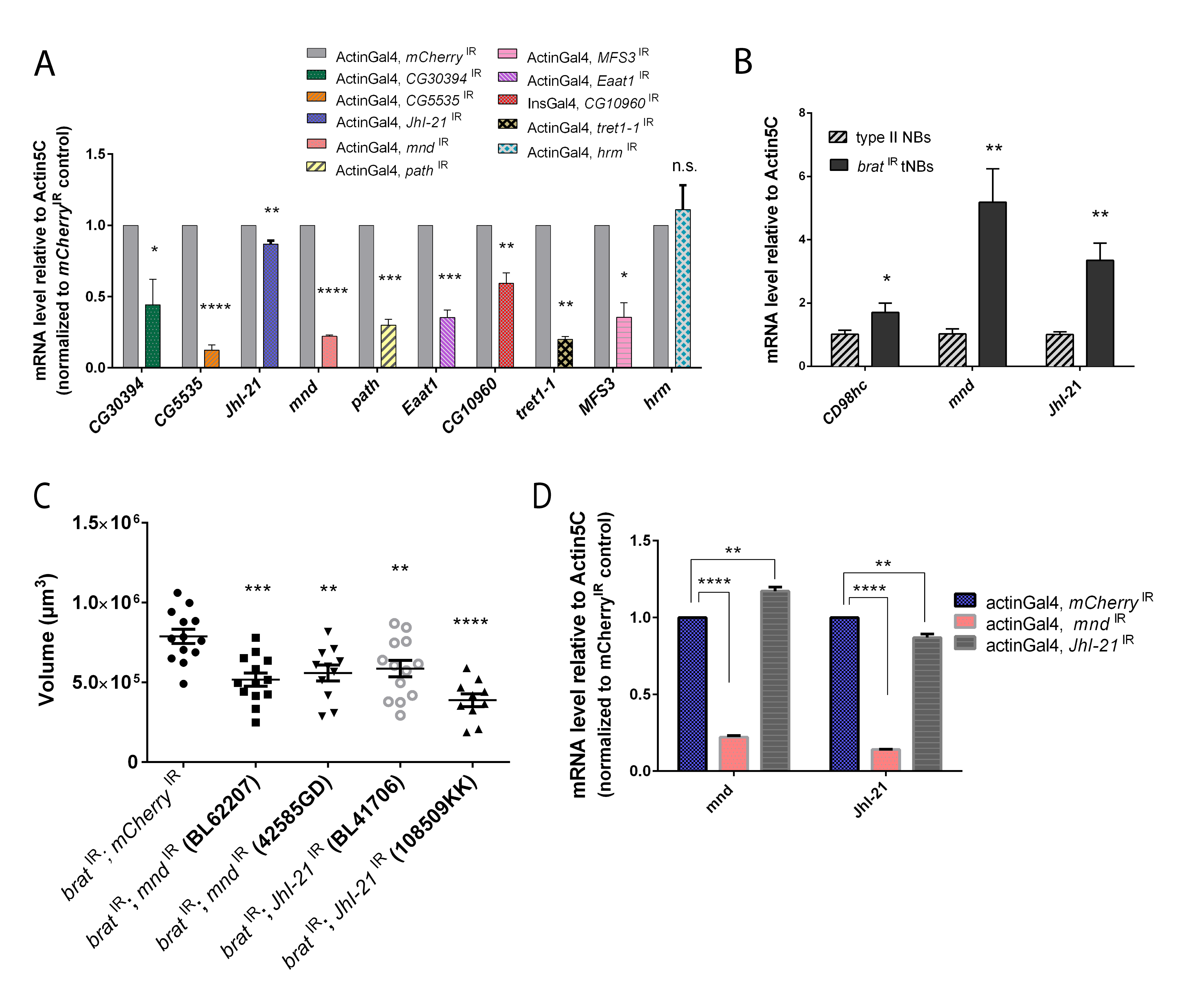

Supplement: Supplementary file 2 — Supplementary file2 Fig. S1 CD98 light chains’ knockdown reduces tumor growth. (A) Quantification of RNAi efficiencies by qPCR. qPCR of indicated target gene in whole brains expressing indicated RNAi transgenic line under the control of actinGal4 except CG10906 IR which was driven by inscGal4. (B) qPCR of CD98hc, mnd and JhI-21 in wild-type type II NBs vs. brat IR tNBs. (C) Quantification of brat IR tumor volumes with individual knockdown of the indicated light chain under the control of type II NB driver PntGal4 with two independent RNAi lines: mnd IR (BL62207 and 42585GD) and JhI-21 IR (BL41706 and 108509KK). Error bars represent ±SEM. Significance for each candidate RNAi compared with control tumors (brat IR; mCherry IR) using a one-way ANOVA with post-hoc Dunnett’s multiple comparisons test. ** P value < 0.01; *** P value < 0.001; **** P value < 0.0001 (D) qPCR of mnd and JhI-21 in control brains (ActinGal4; mCherry IR), mnd knocked down brains (ActinGal4; mnd IR) and JhI-21 knocked down brains (ActinGal4; JhI-21 IR). All RNAi transgenes driven under the control of ubiquitous driver ActinGal4. Whole 3rd instar larval brains were analysed. All RT-qPCR data shown represents the mean (± SEM) of triplicates. Statistical analysis was done using unpaired two-tailed t test. * P value < 0.05; ** P value < 0.01; *** P value < 0.001; **** P value < 0.0001. (TIF 58437 KB) [file 18_2022_4668_MOESM2_ESM.tif]

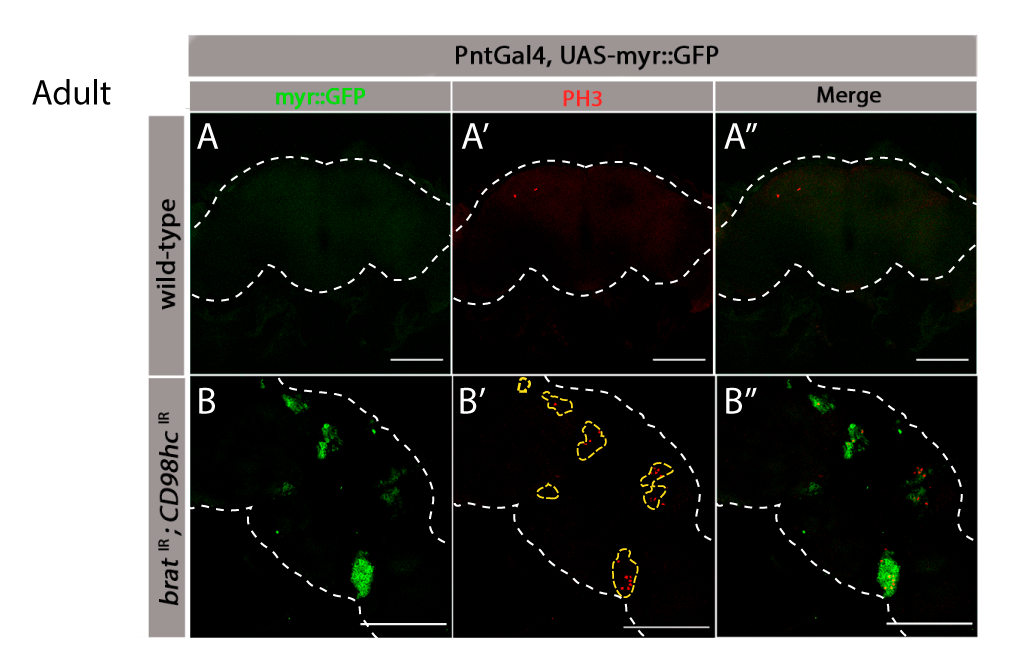

Supplement: Supplementary file 3 — Supplementary file3 Fig. S2 CD98hc depletion does not prevent the immortal proliferative potential of brat IR tNBs. (A-B) Fixed newly ecloded adult brains expressing myr::GFP under the control of type II NB driver PntGal4. (A) Wild-type adult brain (PntGal4, UAS-myr::GFP), (B) brat IR; CD98hc IR tumor (brat IR/CD98hc IR; PntGal4, UAS-myr::GFP). Red, PH3; green, myr::GFP. White dashed lines represent the brain outline and yellow dashed line in B’ represents the tumor outline. Scale bars represent 100 μm (TIF 2480 KB) [file 18_2022_4668_MOESM3_ESM.tif]

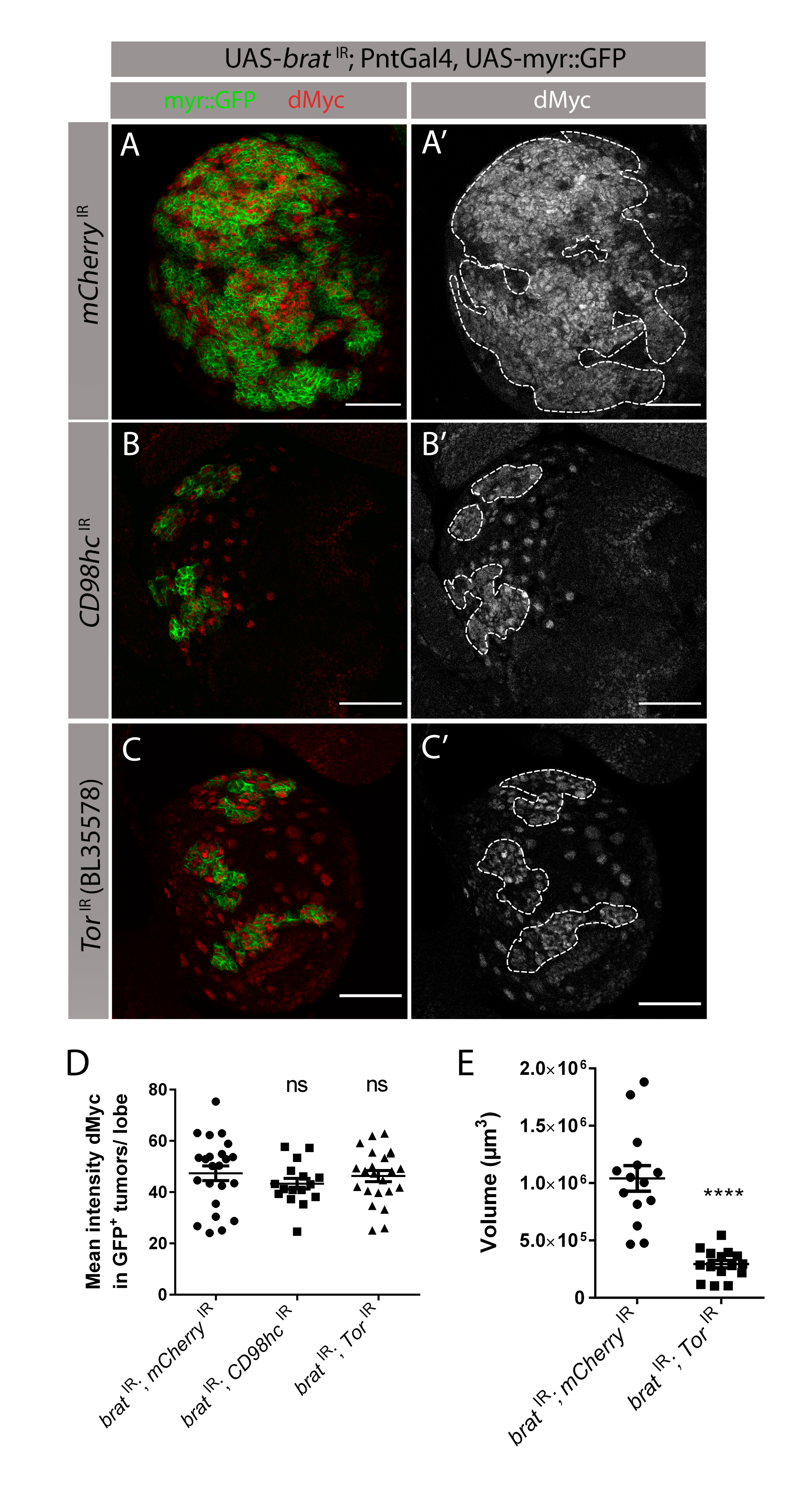

Supplement: Supplementary file 6 — Supplementary file6 Fig. S5 dMyc levels do not change significantly with Tor or CD98hc knock down in brat IR tumors. (A-C) Fixed L3 brain lobes expressing myr::GFP and the indicated transgenes under the control of type II NB driver PntGal4 stained for dMyc (red). myr::GFP, green. (A,A’) control brain tumor (brat IR; mCherry IR), (B,B’) brat IR; CD98hc IR and (C,C’) brat IR; Tor IR tumors. Dashed lines represent the respective GFP+ tumor outline. Scale bars represent 50 μm. (D) Quantification of dMyc mean fluorescence intensity in the GFP+ tumor area. Error bars represent ±SEM. Values for each genotype compared with control tumor (mCherry IR) using a one-way ANOVA with post-hoc Dunnett’s multiple comparisons test. ns – non-significant (P value ≥ 0.05). (E) Quantification of the tumor volume of brat IR; Tor IR tumors in relation to brat IR; mCherry IR control tumors. Statistical analysis was done using unpaired two-tailed t test; **** P value < 0.0001 (TIF 57038 KB) [file 18_2022_4668_MOESM6_ESM.tif]
